# Nucleosome positions alone can be used to predict domains in yeast chromosomes

**DOI:** 10.1073/pnas.1817829116

**Published:** 2019-08-15

**Authors:** Oliver Wiese, Davide Marenduzzo, Chris A. Brackley

**Affiliations:** ^a^School of Physics and Astronomy, University of Edinburgh, Edinburgh EH9 3FD, United Kingdom

**Keywords:** chromatin domains, polymer simulations, MicroC

## Abstract

We use molecular dynamics simulations based on publicly available micrococcal nuclease sequencing data for nucleosome positions to predict the 3D structure of chromatin in the yeast genome. Our main aim is to shed light on the mechanism underlying the formation of chromosomal interaction domains, chromosome regions of around 0.5 to 10 kbp which show enriched self-interactions, which were experimentally observed in recent MicroC experiments (importantly these are at a different length scale from the 100- to 1,000-kbp–sized domains observed in higher eukaryotes). We show that the sole input of nucleosome positioning data is already sufficient to determine the patterns of chromatin interactions and domain boundaries seen experimentally to a high degree of accuracy. Since the nucleosome spacing so strongly affects the larger-scale domain structure, we next examine the genome-wide linker-length distribution in more detail, finding that it is highly irregular and varies in different genomic regions such as gene bodies, promoters, and active and inactive genes. Finally we use our simple simulation model to characterize in more detail how irregular nucleosome spacing may affect local chromatin structure.

## Significance

DNA is packaged into chromosomes, which are further organized into domains: Regions of the genome which are more likely to self-interact. Domains have been observed in species ranging from bacteria to humans and are thought to play an important role in gene regulation. Yet the mechanisms of domain formation are not fully understood. Here we use computer simulations to investigate domain formation in yeast. Our model reproduces the experimentally observed domains using only nucleosome positioning information as an input, implying that (unlike in higher eukaryotes) domain boundary locations are largely determined at this level. Our results reveal how irregular nucleosome spacing impacts the 3D chromosome organization, pointing to a direct link between nucleosome positioning and genome regulation at the large scale.

Recent advances in next-generation sequencing (NGS) technologies have revolutionized our understanding of how the spatial organization of genomes within the cell nucleus impacts gene regulation and cell function. Specifically, the chromosome conformation capture (3C) family of methods gives information about interactions between different chromosome regions (i.e., within a population of cells, how likely are 2 loci to be spatially proximate). High-throughput variants of the method such as Hi-C have shown that most genomes [ranging from bacteria (1) to mammals (2, 3)] are organized into domains, where regions within the same domain are more likely to interact with each other than with regions in different domains. These are usually known as either chromosomal interaction domains (CIDs) or topologically associated domains (TADs). As the sequencing depth of Hi-C data has increased, allowing interactions to be probed at higher resolutions, domains have been found at many different length scales. In mammals, the highest-resolution data have revealed TADs ranging in size from 40 kbp to 3 Mbp (4), and analysis of interactions between neighboring TADs revealed cell-specific “metaTADs” (5); this points to a hierarchical domain structure (6), with domains observed at many scales. The functional role of domains is only just beginning to be understood: Domain boundaries have been shown to provide insulation between enhancers and promoters, which is particularly important for developmental genes (7); disruption of boundaries can lead to misregulation of genes (8); and large-scale rearrangement of TADs has been implicated in diseases such as cancer (9).

Recently a genome-wide chromosome conformation capture method called MicroC (or MicroC XL), developed by Hsieh et al. and detailed in refs. 10 and 11, has allowed chromatin interactions to be probed at the nucleosome level. The technique uses a protocol where chromatin fragmentation is achieved by micrococcal nuclease (MNase) digestion and has yielded nucleosome resolution interaction maps of the entire genome of the budding yeast *Saccharomyces cerevisiae*. At this higher resolution, Hsieh et al. (10, 11) were able to identify CIDs with sizes between 0.5 and 10 kbp or 4 to 50 nucleosomes. These typically contain between 0 and 8 genes, and their boundaries are associated with nucleosome-depleted regions (NDRs) (often found at gene promoters) as well as enrichment of histone modifications associated with transcriptional activity, chromatin remodeling factors, and the cohesin loading factor Scc2 (10). Conventional Hi-C using restriction enzymes to fragment the DNA (12, 13) provides interaction maps with bin sizes down to several kilobase pairs in size (e.g., 5-kbp bins for yeast in ref. 12) so it is unsurprising that the 0.5- to 10-kbp domains were not observed in those data. The latest Hi-C datasets for yeast also do not show a domain structure at larger length scales where they are observed in higher eukaryotes [although earlier Hi-C experiments on unsynchronized cells did show domains with an average size of 200 kbp which correlated strongly with replication timing (14)].

In this paper we use computer simulations based on polymer models to study the formation of domains in yeast. Our aim is to understand how these domains are formed and what determines their boundaries. In other words, we want to understand the essential model ingredients which are required to yield the domain patterns observed in the MicroC data of refs. 10 and 11. Intriguingly, we discover that a deceivingly simple polymer model which includes only the nucleosome positions as an input can already predict many features of the 3D organization to a high degree of accuracy—most notably the locations of domain boundaries. Surprisingly, we find that a model with a more detailed DNA–nucleosome geometry does not in fact show significant differences or improved agreement with the data. This suggests that the information which encodes for 3D domain structure is already present within the map of nucleosome positions. More specifically, we find that it is the irregular spacing of nucleosomes in yeast chromatin which leads to boundary formation. Although nucleosome mapping data showing this irregular spacing have been available for some time, the textbook picture of a regular fiber is still prevalent. To better understand how nucleosome spacing genome-wide differs from a regular fiber we examine the distribution of linker lengths at domain boundaries and in different genomic environments (i.e., within active and inactive genes). We find that the linker-length distribution shows peaks at short, medium, and long ranges, and these are distributed differently in active and inactive regions. By analyzing our simulated chromatin structures, we find that the local compaction of fibers with irregular spacing, such as those constituting the yeast genome, is highly heterogeneous and very much unlike that of regular fibers such as those reconstituted in vitro.

## Results

### A Simple Nucleosome-Level Model for Chromatin.

**Fig. 1.**
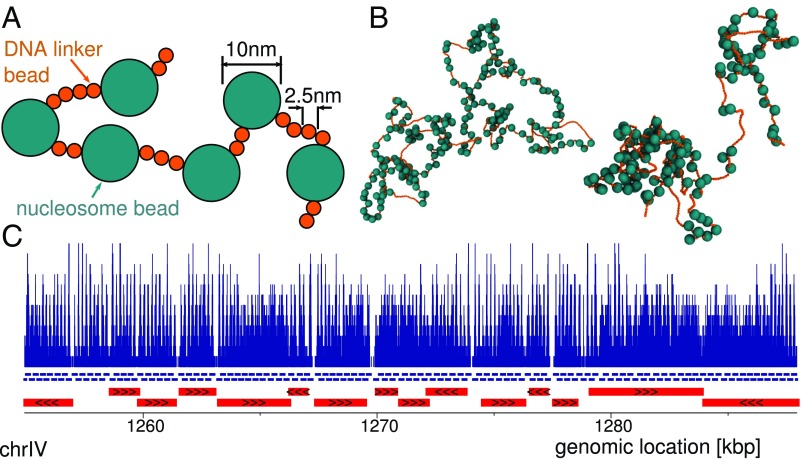
A simple chromatin model using nucleosome positions as input. (*A*) Schematic showing the bead-spring polymer model for a chromatin fiber. DNA is represented as a chain of 2.5-nm beads connected by springs, and we include a bending rigidity to give a persistence length $l_{p}=50$ nm. Nucleosomes (including both histone proteins and wrapped DNA) are represented by 10-nm spheres. (*B*) Typical snapshots from simulations (shown at different zoom levels). (*Left*) Simulation of region chrIV:1254937–1287938 of the yeast genome (SacCer3 build). (*Right*) Simulation of region chrXI:86225–108599. (*C*) Data used as an input to the model. (*Top*) Pile-up of reads from yeast MNase data (from ref. 15) for the indicated genomic region. Blue lines under the plot show the positions of nucleosomes determined using the NucPosSimulator software (16); to aid visualization these are shown in 2 rows with alternating nucleosomes on the top/bottom row. Red bars show genes. Similar plots for other genomic regions are shown in *SI Appendix*, Fig. S1.

We start with the simplest possible model for chromatin which resolves individual nucleosomes and linker DNA. Using a bead-and-spring polymer modeling approach, DNA is represented as a semiflexible polymer where 2.5-nm beads correspond to ∼8 bp of DNA. This is a well-studied model (17–19) and uses simple phenomenological interaction potentials to give the correct bending rigidity for DNA in vivo. Specifically the DNA has a persistence length $l_{p}=50$ nm (this is a measure of the bending stiffness, defined as the distance along the molecule over which correlations in backbone orientation decay). Nucleosomes (including both histone proteins and wrapped DNA) are represented by 10-nm beads. A chromatin fiber is then modeled as sections of DNA (linkers) interspersed with nucleosome beads. For simplicity we do not include any orientational or bending constraints between linkers and nucleosomes; i.e., where there is a connected chain of DNA–nucleosome–DNA beads this acts as a freely rotating joint. We do not include any interactions between nucleosomes or between nucleosomes and DNA, other than simple excluded volume. A schematic of the model is shown in Fig. 1*A*, and full details of all interaction potentials and parameters are given in *SI Appendix*. In the past, several different coarse-grained models for chromatin have been used to study the yeast genome. Some have resolved individual nucleosomes (20–24), while others have studied nuclear organization with much lower-resolution representations of the chromatin fiber, allowing whole nuclei to be simulated (25, 26). The level of detail considered in this work sits between that of these 2 previous approaches. This allows us to simulate large chromosome regions spanning multiple domains, while resolving their internal structure. Importantly, and unlike most previous work, we also include realistic nucleosome spacing, which, as we detail below, plays a key role in determining domain features.

To simulate a specific genomic region, we use MNase digestion followed by sequencing (MNase-seq) data (15) to infer the nucleosome positions and linker lengths. Like the MicroC protocol, these experiments use micrococcal nuclease to digest any DNA which is not protected by nucleosomes, but rather than interaction data they provide a genome-wide map showing nucleosome coverage within a population of cells (Fig. 1*C*, *Top*). Although in reality the exact nucleosome positions will vary from one cell to the next, here for simplicity we considered a single set of “most likely” nucleosome positions (although see *Discussion* and *SI Appendix* for details of simulations which do treat cell-to-cell variation in nucleosome positions). To extract the nucleosome positions from the MNase data we used a software tool called NucPosSimulator (16) as detailed in *SI Appendix* (other tools give similar results; *SI Appendix* and *SI Appendix*, Fig. S2). In Fig. 1*C* these positions are shown with blue lines under the plot; the mean nucleosome spacing is consistent with that quoted previously in the literature (*SI Appendix*, Fig. S2*C*). Importantly these are the only data which are used as an input to the model.

We use the LAMMPS molecular dynamics software (27) to perform Langevin dynamics simulations (see *SI Appendix* for full details). We selected 8 regions of between 15 and 43 kbp long across 6 different yeast chromosomes to get a representative sample of genic chromatin regions. In total our simulations cover ∼240 kbp. After suitable equilibration, we evolve the dynamics to obtain a set of chromatin conformations for each region. (Typically after a 122-τ equilibration simulation, we simulate for a further 50 $\times 10^{3}$ τ and save configurations every 250 τ. We repeat this 20 times for each region, resulting in a population of 2,000 configurations per region, which we find is a representative sample of the equilibrium ensemble (*SI Appendix*, Fig. S3). Here τ is the simulation time unit, equivalent to 80 μs—see *SI Appendix* for full details.) Then, from each population of simulated chromatin conformations we generate a simulated MicroC map. This is done using a stochastic algorithm which mimics the experiment: In short, 2 nucleosomes are picked at random and are said to be in contact with a probability which is a function of their separation. This function includes a length-scale parameter $l_{c}$ (effectively this sets the 3D separation at which 2 nucleosome are deemed to be interacting; full details are given in *SI Appendix* and below). Specifically this generates a map of nucleosome–nucleosome interactions. We compare these maps with MicroC and MicroC XL data from refs. 10 and 11, respectively; in terms of domains these 2 datasets are very similar so we present comparisons to MicroC data here, and below we detail some differences between the datasets. To compare the simulations with the experimental data, we take the data and map each interaction to a specific pair of nucleosomes using the same set of nucleosome positions as in the simulations. This means that (unless otherwise stated) all interaction maps are shown at a nucleosome level and not in base pair coordinates (as is common in Hi-C). Importantly we note that since the MicroC data are not used as an input to the simulations, this is a truly predictive model where 1D nucleosome positioning data are used as an input, and simulated MicroC is the output.

### Nucleosome Spacing Predicts Chromosomal Interaction Domains.

Although this model is simple, as it treats nucleosomes as spheres, rather than a more realistic disk-like shape, and it ignores the complex internucleosome interactions mediated by histone tails, surprisingly we find that it captures sufficient detail to correctly predict many features of short-range nucleosome contacts in 3D.

**Fig. 2.**
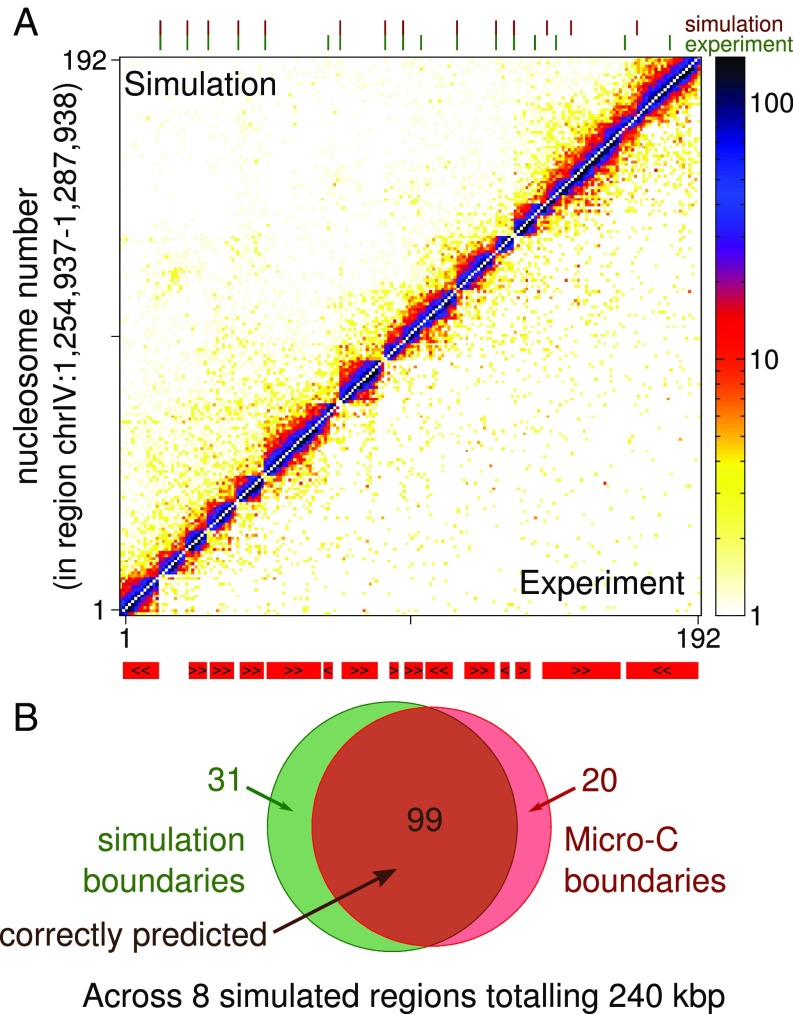
Chromatin fiber simulations accurately predict MicroC interactions for *S. cerevisiae*. (*A*) Map showing interactions between nucleosomes in region chrIV:1254937–1287938. Nucleosomes within the region are numbered starting from 1. The color of the square at coordinates i,j indicates the number of MicroC reads corresponding to interactions between nucleosomes i and j. *Lower triangle* shows MicroC data from ref. 11, while *Upper triangle* shows simulation results. The locations of genes (mapped to the nearest nucleosomes) are shown below the plot (red bars; gene orientation is indicated with black arrowheads). Domain boundaries were called from each map (see *SI Appendix* for details), and these are indicated with tick marks above the plot (upper row shows simulation boundaries in red, and lower row shows experimental boundaries in green). Similar plots for all 8 simulated regions are shown in *SI Appendix*, Fig. S4, with the MNase data used as input in *SI Appendix*, Fig. S1. A comparison between simulations and MicroC XL data is shown in *SI Appendix*, Fig. S9. (*B*) Venn diagram showing the number of domain boundaries across all 8 simulated regions. Overall ∼83.2% of boundaries were correctly predicted by the simulations; an additional 30 boundaries not present in the data were found in the simulated interaction maps. A “correct prediction” is defined as a boundary in the simulated map being within 1 nucleosome of a boundary in the experimental map.

Fig. 2*A* shows results from a simulation of a 33-kbp region of the yeast genome (chrIV:1254937–1287938); a snapshot of a typical conformation for this region is shown in Fig. 1*B*, *Left*. The nucleosome–nucleosome interaction map shows simulation results in Fig. 2*A*, *Upper triangle* and the corresponding MicroC data from ref. 11 in Fig. 2*A*, *Lower triangle* (the simulated map is constructed such that the total number of reads is the same as in the data). First, we note the striking visual similarity between the 2 maps, especially close to the diagonal. Second, to more quantitatively compare the simulations with the experiments, we identified domains by calling boundaries (see *SI Appendix* for details). In this region the MicroC data show 17 boundaries; our simulations correctly predict the location of 11 of these (64%). As well as the correctly predicted boundaries, the simulations also predict an additional 3 boundaries which are not found experimentally. Extending this analysis to all 8 simulated regions, which cover a total of 240 kbp (Fig. 2*B*), we find that remarkably the simulations correctly predict the positions of 83.2% of boundaries (99 of 119; by comparing this to randomly generated domains with the same mean size we find that the p value for this level of agreement is less than $10^{-11}$; *SI Appendix*), but also predicted 31 additional boundaries (i.e., 76.0% of simulation boundaries were correct). A more local measure of chromatin interactions is the “insulation signal,” which quantifies interactions between regions on opposite sides of a given nucleosome (*SI Appendix*); comparing the simulated and MicroC insulation signals we find a correlation coefficient r=0.62 ($p<10^{-10}$ using the Spearman rank correlation; *SI Appendix*, Fig. S5*A*). If we consider the insulation signal only for boundaries, the correlation increases to r=0.76 ($p<10^{-10}$; *SI Appendix*, Fig. S5*B*).

In summary, in terms of boundary prediction and the striking visual agreement between contact maps, our model performs surprising well given its simplicity. Since the only input data to the model are nucleosome positions, we conclude that they are a major driver of chromatin interactions at this scale. One might expect that a pair of widely spaced nucleosomes could act as a boundary to nucleosome interactions. Indeed the nucleosome spacing (or linker lengths) at boundaries tends to be much larger than average (within the simulated regions boundary linkers are on average ∼117 bp, compared to ∼28 bp for all linkers; *SI Appendix*, Fig. S6*A*). One might consider that since MicroC implicitly gives only interaction information from nucleosomes, then the observation of boundaries at large linkers (NDRs, where there is no MicroC signal) could be an artifact. To check that this is not the case we generate a contact map where interactions are sorted into regularly sized bins (*SI Appendix*, Fig. S7 *A* and *B*) and normalized for variation of nucleosome occupancy (i.e., taking NDRs into account). Further, since our simulations give the full chromatin configuration (including nucleosomes and linker DNA), we can generate a map which includes contacts from all regions of the fiber (*SI Appendix*, Fig. S7*C*). Both of these maps show domains, confirming there is a real effect of increased self-interaction within those regions.

Examining the boundaries found in the simulations in more detail, we find that the 20 “missing” boundaries (i.e., those present in the MicroC data but not found in simulations) tend to be at more closely spaced nucleosomes (short linkers, on average ∼25 bp; *SI Appendix*, Fig. S6*B*). Using data for histone modifications and protein binding (from refs. 28 and 29, respectively) we find that the correctly predicted boundaries tend to be flanked by nucleosomes enriched in marks associated with gene activation (H3K9, H3K18, and H3K56 acetylation and H3K4me3; *SI Appendix*, Fig. S6*C*), consistent with the findings of refs. 10 and 11. Interestingly, the missing boundaries lack any significant enrichment of these marks (i.e., they do not display the features found at most boundaries), and they tend to be weaker (see *SI Appendix* for details of boundary strength quantification). Together this suggests that the missing boundaries might not in fact be real boundaries, but rather incorrect calls which the simulations correctly fail to reproduce.

The 31 “extra” boundaries which are present in simulations but not found in the MicroC data were found to be flanked by nucleosomes depleted in most “active” histone modifications, but enriched in H3K36me3 (*SI Appendix*, Fig. S6*C*). It was noted in ref. 10 that H3K36me3 is highly depleted at MicroC boundaries—together with the results presented here this suggests that this mark is associated with a mechanism which promotes nucleosome–nucleosome interactions across a long linker (or NDR) which would otherwise act as a boundary. In yeast, dimethylation and trimethylation of H3K36 have been associated with transcription (30), and the Set2 enzyme responsible for generating these marks is thought to interact with RNA PolII in a way which is consistent with cotranscriptional H3K36 methylation (31). While ∼82% of MicroC boundaries within the simulated regions are at, or near to, gene promoters, ∼77% of the extra boundaries are within gene bodies (none are at promoters). Together this suggests that transcription is another important determinant of boundaries: Long linkers (which are normally associated with promoters) are natural boundaries, except when they occur within a gene body, in which case active transcriptional elongation appears to abrogate the boundary.

### Effect of Cross-Linker Length on Interaction Maps.

**Fig. 3.**
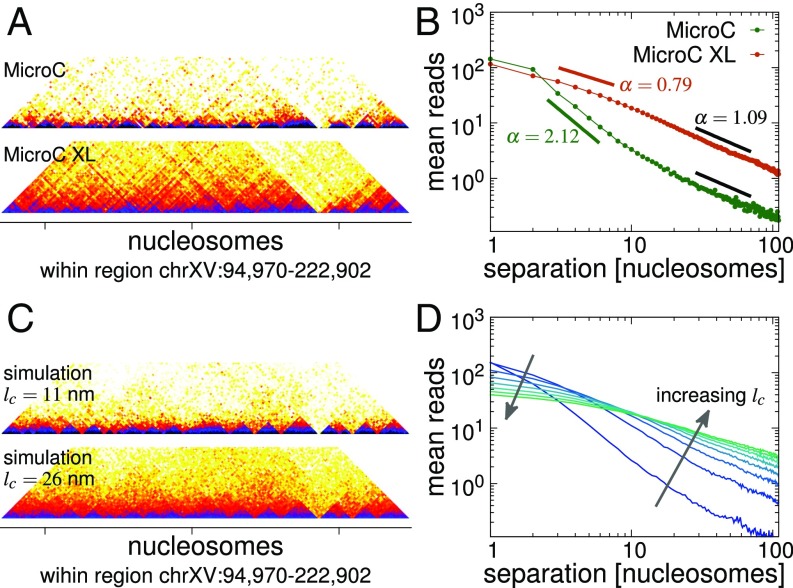
MicroC XL data show similar domains, but how the number of reads decays as a function of genomic separation differs. (*A*) Plot showing interaction maps (rotated through 45° and cropped) for the same region of chrXV for MicroC (*Top*) and MicroC XL (*Bottom*) data from refs. 10 and 11, respectively. The same domains appear in both plots, but the level of interactions differs for larger separations (*Bottom* map shows darker colors toward the top). (*B*) Plot showing how, on average, the number of interaction reads between nucleosomes scales with their genomic separation for the 2 experimental datasets (measured in nucleosomes; i.e., a separation of 1 means neighboring nucleosomes). A straight line on a log−log plot indicates a power law, and exponents in different regions are indicated. The points represent an average over all simulated regions. (*C*) Interaction maps from simulations of the same genome region as in *A*. The 2 maps are obtained from the same set of simulations, but a different “cross-linking” length scale $l_{c}$ is used to generate the map. Values of $l_{c}=11.25$ nm and $l_{c}=26.25$ nm give the best fit to the MicroC and MicroC XL data, respectively (*SI Appendix*). (*D*) Mean reads vs. separation plot from simulations (average over all simulated regions). Each line is obtained from interaction maps with different values of $l_{c}$ ($l_{c}=7.5,15,22.5,30,37.5,45,52.5$, and 60 nm).

Although the MicroC data published in ref. 10 revealed a short length-scale interaction domain pattern, the method could not recapitulate higher-order features observed in conventional Hi-C (e.g., centromere or telomere interactions characteristic of the Rabl configuration). It was later found that the discrepancy arises because the cross-linking agent formaldehyde is able to cross-link nucleosomes only at a very short distance, and an improved version of the method, named MicroC XL, using 2 different, longer, cross-linkers was developed (11). Data from MicroC XL experiments showed both domains and higher-order chromatin interactions; while the domain pattern is largely unaffected, a striking difference between MicroC and MicroC XL data is in how the number of interactions depends on the genomic separation, s. This is evident in the interaction maps (Fig. 3*A*) and can be shown explicitly via a plot of the mean number of interactions for a given (nucleosomal) separation (Fig. 3*B* and *SI Appendix*, Fig. S8). Similar plots from other experimental methods often show a power-law relationship ($\text{mean number of reads}∼s^{-\alpha }$ over several decades in s), which is indicated by a linear relationship on a log−log plot. Here both the MicroC and MicroC XL data show linear regions, although interestingly, there seem to be different power-law regimes for short-range (separation s≈2 to 10 nucleosomes) and long-range (s≈10 to 100 nucleosomes) interactions. The difference between the regimes is much stronger for the MicroC than for the MicroC XL data, and both datasets show a similar slope in the 10- to 100-nucleosome range (Fig. 3*B* shows experimental data for the genomic regions we simulated; a discussion of the genome-wide scaling is given in *SI Appendix* and *SI Appendix*, Fig. S8 *A* and *B*).

The main difference between the MicroC and MicroC XL protocols is the length of the cross-linkers; we reasoned that this could be accounted for in our simulated interaction maps by changing the length-scale $l_{c}$ which controls how the map is built from the simulated chromatin configurations (one can think of this as a “cross-linker length scale”). Fig. 3*D* shows that indeed by increasing $l_{c}$ we go from a curve which is closer to the MicroC data to one which is closer to the MicroC XL data (*SI Appendix*, Fig. S8 *C* and *D*). Importantly the positions of the domain boundaries remain largely unaffected (*SI Appendix*, Fig. S9); using a value of $l_{c}$ which results in a reads vs. separation curve which best fits the MicroC XL data, we find that ∼84% of boundaries found in the MicroC XL data were also present in the simulations and ∼69% of simulation boundaries were correct (*SI Appendix*, Fig. S9*H*).

The universal scaling exponents for the power-law decay of polymer interaction probability as a function of contour length have been well studied in the polymer physics literature (32). The fact that the apparent exponents we observed depend on the cross-linking agent used (33) suggests that we have not yet reached the asymptotic regime of large s for which the exponent is universal. For example, in our simulations we would expect an asymptotic power-law decay with exponent α∼2.18 as befits a self-avoiding chain (34). By fitting a power law to the s=10- to 100-nucleosome range for our simulations we find that α decreases with $l_{c}$ (*SI Appendix*, Fig. S8*D*, *Inset*). In summary, we conclude that care must be taken when equating exponents measured from 3C-based experiments with polymer critical exponents, since the former may depend on the exact experimental protocol.

### A Nucleosome Model with a More Detailed Geometry Does Not Improve Domain Predictions.

**Fig. 4.**
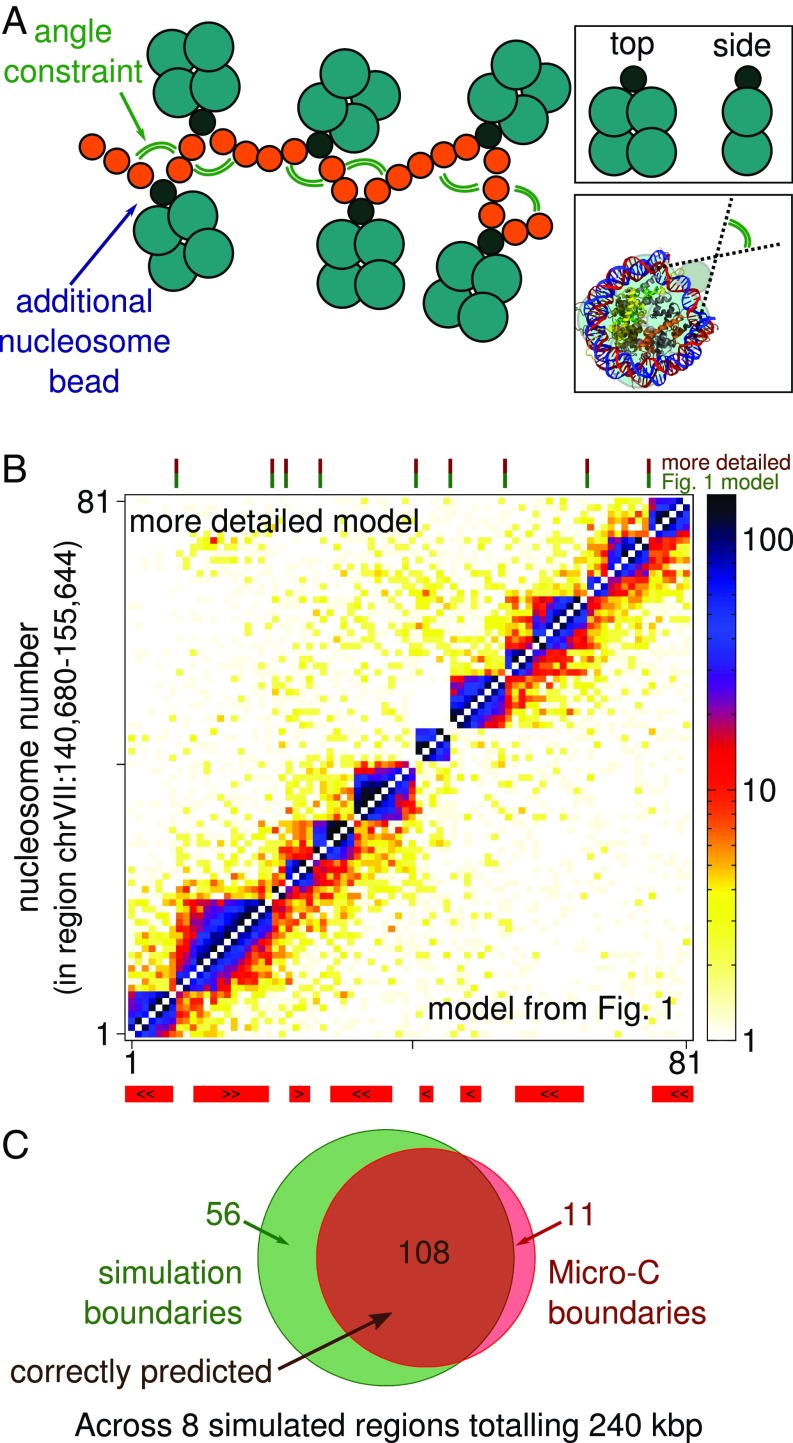
A more detailed model takes into account known aspects of nucleosome geometry. (*A*) Schematic showing the more detailed model. Nucleosomes are made of 5 beads, diffusing as a single object. Four larger beads arranged in a square approximate the disk shape of a nucleosome, with diameter roughly 10 nm and height 5 nm. The linker DNA is attached to the nucleosome through a smaller bead at 1 edge of the nucleosome, mimicking the way the entry/exit linkers leave the nucleosome on the same side at a preferred angle. (*A*, *Top Inset*) Top and side views show a more disk-like nucleosome shape. (*A*, *Bottom Inset*) The nucleosome schematic is overlaid on an image of the nucleosome crystal structure (obtained from PDB: 1KX5, ref. 35) to show the preferred linker exit/entry angle. (*B*) Interaction map for region chrVII:140680–155644, where *Lower triangle* shows simulations using the model described in Fig. 1 and the *Upper triangle* uses the more detailed model shown in *A*. (*C*) Venn diagram comparing boundaries predicted with the more detailed model and those obtained from the MicroC data.

As noted above, it is surprising that our simple model can give such a good prediction of nucleosome interactions at the domain level. We might expect that a more detailed representation of the nucleosome geometry, which is well known from crystallography (36, 37), may be an important aspect to include and that it might increase the agreement with chromatin interaction data. We therefore now turn to a model where 1) we use a more realistic “disk-like” shape for the nucleosomes instead of a 10-nm sphere and 2) we simulate the way linker DNA enters/exits the nucleosome by including an angle constraint (24). A schematic is shown in Fig. 4*A*. The more detailed description possesses some (but not all) of the features included in the highly detailed models described in refs. 20 and 22, which have been used in Monte Carlo simulations to study the folding of short arrays of regularly spaced nucleosomes into 30-nm fibers.

Intriguingly, despite the improved geometrical resolution of the nucleosomes, there is no appreciable improvement in agreement with the MicroC data. In Fig. 4 *B* and *C* we show results for the version of the model where, as for the simpler model, there are no nucleosome–nucleosome interactions except for volume exclusion. Visually the interaction maps look very similar to those generated by the model in Fig. 1. Comparing boundary calls, this model correctly identifies more boundaries: 90.8% of experimental boundaries were found, compared to 83.2% for the model in Fig. 1. However, it also shows more extra boundaries: 65.9% of simulated boundaries are correct, compared to 76.0% for the model in Fig. 1. The correlation between the MicroC and simulation insulation signal is r=0.52 ($p<10^{-10}$), about 16% smaller than for the model in Fig. 1. And finally, there is a marked difference between the 2 models in terms of how the average interactions vary with separation (*SI Appendix*, Fig. S10): At separation s>20 nucleosomes the more detailed model deviates significantly from the MicroC data.

Another detail which can be added to the model is the presence of attractive interactions between nucleosomes [which might be mediated by surface charges on the nucleosome core and/or the histone tails (38)]. This is included in the model described in Fig. 4*A* in a simple way, by introducing an attractive interaction between the center points of each nucleosome (for details see *SI Appendix*). As expected, this leads to an increase in nucleosome–nucleosome contacts and, depending on the interaction energy $\epsilon_{n}$, can particularly promote long-range interactions (*SI Appendix*, Fig. S11). We note though that in all cases the model still fails to show better agreement with either the MicroC or the MicroC XL data compared to the simple model. For this reason, for the rest of this work we return to the simpler model in Fig. 1.

### Nucleosome Spacing Is Irregular in Yeast Chromatin Genome-Wide.

Visual inspection of the simulated chromatin conformations we generated (Fig. 1*B*) shows that nucleosome spacing is highly irregular, and this leads to the formation of a heterogeneous fiber. Although nucleosome positioning data have been available for some time, this fact is often overlooked in discussions of the formation of chromatin fibers (as typical textbook pictures usually show regular spacing). We now ask whether irregular nucleosome spacing is a generic feature of yeast chromatin in vivo, and we examine nucleosome spacing genome-wide.

**Fig. 5.**
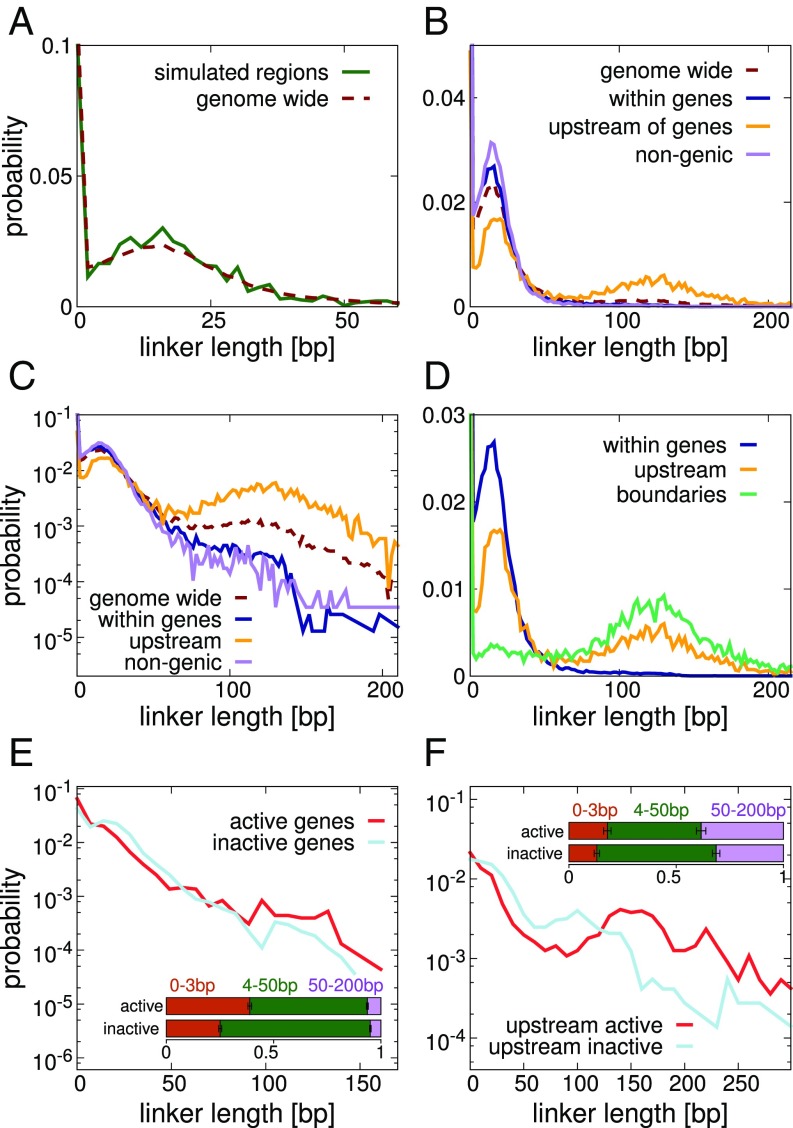
Linker lengths have a multimodal distribution. Plots show the linker-lengths distribution based on nucleosome positions generated by NucPosSimulator (using MNase-seq data from ref. 15). (*A*) The genome-wide distribution (red dashed line) is shown alongside that for the 8 simulated regions (green solid line). (*B*) Separate distributions are shown for nucleosomes within genes (including all annotated genes of length ≥1 kbp), within the 500 bp upstream of gene transcription start sites (TSS) (the same set of genes is used), and in nongenic regions of the genome. (*C*) The same plot as in *B* is shown on log-linear axes. (*D*) The distribution of linker lengths found at domain boundaries (found genome-wide using the same method as described above) is shown alongside the “within gene” and “upstream of gene” distributions. (*E*) Distributions for nucleosomes within active and inactive genes are shown separately. (Activity is inferred from ChIP-on-chip data for PolII, obtained from ref. 39.) *Inset* shows the proportion of linkers with length less than 200 bp which fall into the 3 indicated length ranges, with error bars showing SEs. Nonoverlapping error bars indicate a statistically significant difference. (*F*) Similar plot to that in *E* but for linkers of nucleosomes in the 500 bp upstream of gene TSS.

Fig. 5*A* shows the distribution of linker lengths across the 8 simulated regions; also shown is the genome-wide distribution (nucleosome positions generated using the NucPosSimulator software as before). First, we note the concordance between these distributions, indicating that the simulated regions are representative. Second, we note that the distribution is far from what would be expected for either regularly or randomly spaced nucleosomes. In the former case, one would expect a Gaussian distribution around a mean value; in the latter case, if nucleosomes were positioned by a Poisson process, one would expect an exponential distribution. In fact, the distribution is multimodal, with a large number of very short linkers (about 25% of linkers genome-wide have length 1 to 3 bp) and a broad peak centered on ∼16 bp. Interestingly there are many linkers which are much longer (about 12% of linkers are between 50 and 200 bp), which presumably correspond to NDRs, such as are found at gene promoters. (We assume most linkers greater than 200 bp are artifacts due to unmappable regions of the genome.) Typically the nucleosome repeat length for yeast is quoted as 165 bp (40, 41), which corresponds to a linker length of 18 bp. From the distribution shown in Fig. 5*A*, the mean linker length is ∼28.7 bp (and this decreases to ∼18 bp if only those linkers which are ≤100 bp are considered).

In Fig. 5 *B*–*D* we examine the linker-length distribution more closely by separating out different types of genomic region. Specifically we look at linker lengths 1) within genes, 2) within regions 500 bp upstream of genes (promoters)* , and 3) in nongenic regions. To unambiguously identify linkers within gene bodies, we limit the analysis to genes of length ≥1 kbp in categories 1 and 2, but consider all annotated genes when determining linkers in category 3. We find that linkers within genes and in nongenic regions show a similar size distribution (although there are more short, <3-bp linkers within gene bodies: ∼30% compared to ∼25%). As expected, in the promoter regions there are also many long (50 to 200 bp) linkers (∼40%) and a lower proportion of short- and medium-length linkers. Fig. 5*D* confirms that genome-wide, boundaries tend to be at long linkers (with the adjacent linkers tending to be short or medium in length).

In Fig. 5 *E* and *F* we further separate active and inactive genes using PolII binding data (39) as a proxy for transcriptional activity (we take genes with PolII binding levels below and above the 10th and 90th percentiles, respectively). This reveals that the bodies of active genes have more very short linkers and fewer medium (4 to 50 bp) linkers than those of inactive genes (Fig. 5*E*). This is consistent with previous work (42) which found a correlation between gene activity and nucleosome density within coding regions (suggesting that, perhaps surprisingly, nucleosome crowding strongly facilitates transcription elongation) and proposed that transcriptional plasticity (the variation of gene expression as a result of environmental changes) may be facilitated by chromatin remodelers which alter nucleosome spacing. Other recent work (43) revealed a correlation between nucleosome crowding (i.e., closely spaced or even overlapping nucleosomes) and increased nucleosome turnover, which itself is associated with gene activity (44). The promoter regions of the active genes also showed slightly more short linkers than their inactive counterparts, as well as more long linkers (Fig. 5*F*).

To check that the linker-length distribution present in our simulation is not an artifact of the specific experimental technique used to generate the data (MNase-seq) or the method used to extract nucleosome positions from the MNase data, in *SI Appendix* (also *SI Appendix*, Fig. S12) we present a similar analysis of linker lengths obtained from site-directed DNA cleavage experiments, which offer higher-resolution data than MNase-seq (45).

### Chromatin Conformations with Irregular (Realistic) Nucleosome Spacing Are Heterogeneous and Differ from Regular Fibers.

**Fig. 6.**
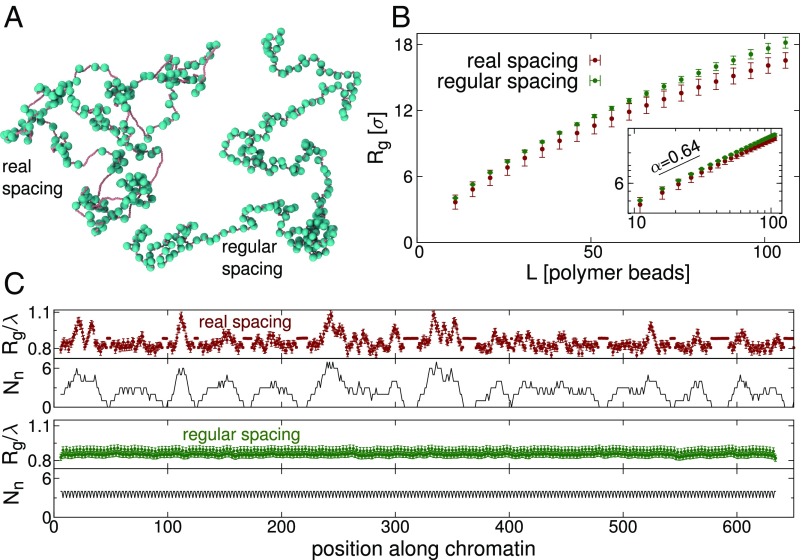
Irregular nucleosome spacing affects the local size and structure of the fiber. (*A*) Snapshots from simulations of (*Left*) yeast genomic region chrIV:1254937–1287938 using nucleosome spacing obtained from MNase-seq data (shown in Fig. 2*C*) and (*Right*) a fiber of similar length with regularly spaced nucleosomes (linker length 22 bp). (*B*) Plot showing the radius of gyration, $R_{g}$, as a function of the length of the polymer (measured in numbers of beads—main text). Lengths are given in units of σ=2.5 nm. Error bars show the SD. Irregular spacing tends to reduce the size of the polymer. *B*, *Inset* shows the same data on a log−log plot: A straight line indicates a power-law relationship ($R_{g}∼L^{\alpha }$). The black line shows the exponent obtained from a fit to the real nucleosome spacing case. (*C*) *Top plots* (colored points) show the average $R_{g}/\lambda$ of an L=11 bead region, as a function of position along the fiber, for the irregular (chrIV:1254937–1287938 region) and regular spaced cases. Here λ is the square root of the contour length within the window in simulation length units ($\lambda =\sqrt{N_{d}+4N_{n}}$). Error bars show the SE in the mean. *Bottom plots* (black lines) show the number of nucleosomes $N_{n}$ within the window.

We now use our simulations to examine some of the properties of 3D structures formed by fibers with irregularly spaced nucleosomes, by comparing these to fibers of similar length with regularly spaced nucleosomes (Fig. 6*A*). First, we ask how nucleosome spacing affects the volume taken up by the chromatin fiber. Fig. 6*B* shows how the radius of gyration ($R_{g}$, a measure of the size of the fiber) varies as a function of fiber length for the 2 cases. We calculate this by finding $R_{g}$ for the first L beads of the fiber (treating DNA and nucleosome beads on the same footing), then beads 2 to L+1, then beads 3 to L + 2, and so on; we average over all such windows of length L and over snapshots taken at intervals during the simulation as before. We find that overall the irregularly spaced fiber is smaller than the regular case ($R_{g}$ reduces by about 10%); this could be interpreted as a decrease in the effective persistence length or stiffness of the fiber. Fitting a power law, we find a similar exponent in each case (α≈0.64 for the irregularly spaced nucleosomes and α≈0.67 for the regular case): These are likely finite N cross-overs to the value expected for large N for a polymer in a good solvent (α≈0.588).

Next, we examine how irregular nucleosome spacing affects the local fiber compaction, again using the radius of gyration as a measure. This time we use a fixed region length of L=11 beads and slide this window along the fiber, calculating at each position $R_{g}$ averaged over different snapshots and repeat simulations. Since the window consists of a mixture of DNA and nucleosome beads, we scale this by a factor $\lambda =\sqrt{N_{d}+4N_{n}}$, where $N_{d}$ and $N_{n}$ are the numbers of DNA and nucleosome beads within the window, respectively (i.e., the square root of the contour length, since nucleosome beads have a size 4 times that of DNA beads). For the irregularly spaced fiber (Fig. 6*C*, *Top*) $R_{g}/\lambda$ varies widely with position along the fiber. The origin of the variation is revealed by a plot of the number of nucleosomes within each window (Fig. 6*C*, black lines). For a window with no nucleosomes we find $R_{g}/\lambda \approx 0.88$^†^ . Adding a small number of nucleosomes to the region effectively introduces turning points into the polymer and so reduces $R_{g}/\lambda$. However, if many nucleosomes are added to the region, the steric interaction between these leads to an effective stiffening of the chain and an increased $R_{g}/\lambda$. For the regularly spaced nucleosomal fiber, the $R_{g}$ profile is, as expected, virtually flat. A regular fiber also yields a nucleosome interaction map which is devoid of domains.

These simulations show that different spacing of nucleosomes leads to relatively small, yet significant, differences in the global and local 3D organization of chromatin.

## Discussion

In this work we have presented a simulation model for chromatin, using it to study interactions within the chromosomes of the budding yeast *S. cerevisiae*. Surprisingly this seemingly simple model, where nucleosomes are represented by 10-nm spheres connected by linker DNA (Fig. 1), had sufficient detail to correctly predict the nucleosome interaction patterns observed in recent MicroC data (10, 11) (which revealed “chromosomal interaction domains” of typical length ∼1 to 2 kbp). Specifically the simulations were able to correctly determine the locations of 84% of domain boundaries in 8 simulated genomic regions across 6 chromosomes. Simulations of a fiber with uniformly spaced nucleosomes did not show domains. Additional microscopic details such as a disk-like nucleosome shape and constraints on the exit/entry angles for linker DNA were not required (i.e., a model including these features did not show any appreciable improvement in the agreement with the data).

Since the only data used as an input for the simulations were the genomic positions of nucleosomes, this implies that the pattern of domain boundaries is largely encoded in these positions. (Indeed the domain pattern can also be predicted using a simple analytical approach—*SI Appendix* and *SI Appendix*, Fig. S13.) While previous work (10) found domain boundaries to be enriched for binding of some proteins, and the flanking nucleosomes were enriched for transcriptional activating histone modifications, our results suggest that these are not directly responsible for boundary formation. Rather, protein binding (e.g., of chromatin remodelers) more likely gives rise to the formation or maintenance of nucleosome-depleted regions, and this in turn forms a boundary. Our model also incorrectly predicted that boundaries would be present at some long linkers within gene bodies—this failure is, however, itself informative, since it suggests that transcription through NDRs can counter their boundary-forming potential. Overall these results suggest that since the domains observed in the MicroC data occur as a consequence of nucleosome positions, they are a “signature” of regulatory mechanisms (domains follow function); this is different from the current understanding of the larger domains in higher eukaryotes, which are thought to control chromatin interactions to regulate expression (function is driven by domains).

In light of the important role of nucleosome spacing in chromatin interactions, we next examined the linker-length distribution in more detail (using both positions generated from MNase data and other experimental methods—*SI Appendix*). The surprising number of very closely spaced or even overlapping nucleosomes and the high abundance of these within (particularly the most active) gene bodies suggest that this has a role in transcription elongation (42).

Finally, we used our simulations to study how the irregularity of linker lengths affected the 3D polymer properties of chromatin fibers. We found that a chromatin fiber made from irregularly spaced nucleosomes leads to an overall reduction in the size of the polymer compared to a regularly spaced fiber of the same length. By examining the local compaction of a region of the chromatin fiber as a function of the position along it, we found that irregular spacing leads to wide variation of 3D size compared to the regularly spaced nucleosomes case. In our model, this variation closely followed the number of nucleosomes within the region. Unexpectedly, we found a nonlinear relationship between the number of nucleosomes within a region and its 3D size—for a small number of nucleosomes the size reduces compared to a region with linker DNA only, whereas if many nucleosomes are present the 3D size is larger. Since the entry/exit angle of the linker DNA (which is not constrained in our simple model) is also likely to have an effect, it would be of interest to study this with a more detailed simulation scheme in future.

In summary, our simulations have revealed a close link between nucleosome positioning and chromatin interactions in 3D in yeast. Although genome-wide data on nucleosome positions have been available for several years, the striking irregularity in nucleosome spacing is often overlooked. It will be of interest to study how this affects the 3D structure of chromosomes at larger length scales (46)—for example, future models could investigate the effect of the torsional rigidity of DNA, which controls the relationship between linker length and the relative orientation of adjacent nucleosomes. One must also bear in mind that there are still many challenges in obtaining nucleosome positions, and the maps generated to date rely on information from a population of cells—it is still unclear what the nucleosome landscape is like within a single cell (47). In the simulations detailed above we considered a set of “most likely” nucleosome positions for each region. In *SI Appendix* we present some simulations which take cell-to-cell variation of nucleosome occupancy into account (*SI Appendix*, Figs. S14 and S15). This was done by using a different set of nucleosome positions based on the same MNase data in each of 20 simulations of the same region. As one might expect, the level of agreement between the simulations and the MicroC data depends on the level of variability between the different input sets of positions. Interestingly input with only a low level of variation between nucleosome positions provided the best agreement with the MicroC data (*SI Appendix*, Fig. S15), suggesting that for yeast, the nucleosome positions are highly conserved across a population of cells. It would be interesting to study this further in the future, as single-cell experimental measures of nucleosome occupancy develop (48). Another important question is how nucleosome positions are determined in the first place. While our simulations involved nucleosomes at fixed positions, in reality they are likely to be more dynamic, with factors such as DNA sequence, the action remodeling complexes and histone chaperones, and transcription and replication playing important roles. These are active areas of research, and it would be interesting to see whether there is any link with domains.

In higher eukaryotes the family of H1 linker histone proteins, which have been shown to induce folding of regularly spaced nucleosomal arrays into 30-nm fibers in vitro, is highly abundant and found across the genome, particularly in heterochromatin [to the contrary, the yeast homologue *HHO1p* has been found not to be present through most of the genome, but rather only at restricted locations (49)]. Our simulation snapshots showing irregularly spaced nucleosomes are strikingly reminiscent of recent imaging experiments in human cells (50) which revealed spatially heterogeneous groups of nucleosomes known as “clutches.” It would be of interest to study irregular nucleosome spacing in that context—how it varies in different genomic regions and what the implications are for higher-order fiber folding—particularly since H1 is thought to control nucleosome repeat length and is found to be depleted near active genes and promoters. Similarly, it would be interesting to understand whether linker length plays a role in domain boundary formation at larger length scales in higher organisms—although chromatin looping and interactions between regions with similar histone modifications have been implicated there (51), long linkers might lead to kinks or distortions in the chromatin fiber which promote certain loops (52), or they might provide a natural barrier to the (1D and 3D) spread of histone modifications (53). This may provide a mechanical link from DNA sequence, through nucleosome positioning, to higher-order chromosome organization.

## Materials and Methods

In this work we perform Langevin dynamics simulation of a chromatin fiber modeled as a bead-and-spring polymer using the LAMMPS software (27). In brief, a fiber which resolves individual nucleosomes is represented by a chain of 2 species of spherical beads. Small (2.5 nm diameter) beads represent linker DNA, while larger (10 nm diameter) beads represent nucleosomes. We use a common model for DNA (17–19) which correctly captures its bending stiffness and includes steric interactions. LAMMPS integrates the Langevin equation for each bead in the simulation using a velocity-Verlet algorithm, where an implicit solvent provides a thermostat which results in a constant NVT ensemble. Full details of the model and simulation scheme are given in *SI Appendix*. For the simulations presented in Fig. 4 the nucleosomes are instead represented as a rigid body composed of 5 smaller beads, arranged to approximate a 10-nm × 5-nm disk, where linker DNA forms an entry/exit angle of $72^{○}$; full details are given in *SI Appendix*.

We compare our simulations to MicroC and MicroC XL data obtained from refs. 10 (GEO:GSE68016) and 11 (GEO:GSE85220), respectively; these were aligned to the *S. cerevisiae* genome (SacCer3 build) following the methods discussed in those references. We further map the MicroC data onto the set of most likely nucleosome positions obtained from MNase-seq data (from ref. 15, GEO:GSM53721), using the NucPosSimulator software (16). Full details are given in *SI Appendix*.

## Supplementary Material

Supplementary File

## Footnotes

*Note that in some cases the region 500 bp upstream of a gene overlaps with the 5′ end of the adjacent gene on the same strand or the promoter region of an adjacent divergent gene.

^†^The persistence length for our model DNA is 20 beads, so following the worm-like chain model in the rigid rod limit we would expect a value not far from the upper bound $R_{g}/\lambda =\left(L/\sqrt{12}\right)/\sqrt{L}\approx 0.96$, in agreement with our data.
