# Supplementary material for: Nucleosome positions alone can be used to predict domains in yeast chromosomes
Source: Proc Natl Acad Sci U S A. 2019 Aug 15;116(35):17307–15. doi: 10.1073/pnas.1817829116 (PMC6717315; doi:10.1073/pnas.1817829116)
Supplement: Supplementary File [file pnas.1817829116.sapp.pdf]

PNAS
www.pnas.org

Supplementary Information for

Nucleosome positions alone can be used to predict micro-domains in yeast chromosomes

O. Wiese, D. Marenduzzo, and C. A. Brackley

Corresponding Author C. A. Brackley.
E-mail: C.Brackley@ed.ac.uk

This PDF file includes:

Supplementary text
Figs. S1 to S15
References for SI reference citations

www.pnas.org/cgi/doi/10.1073/pnas.1817829116

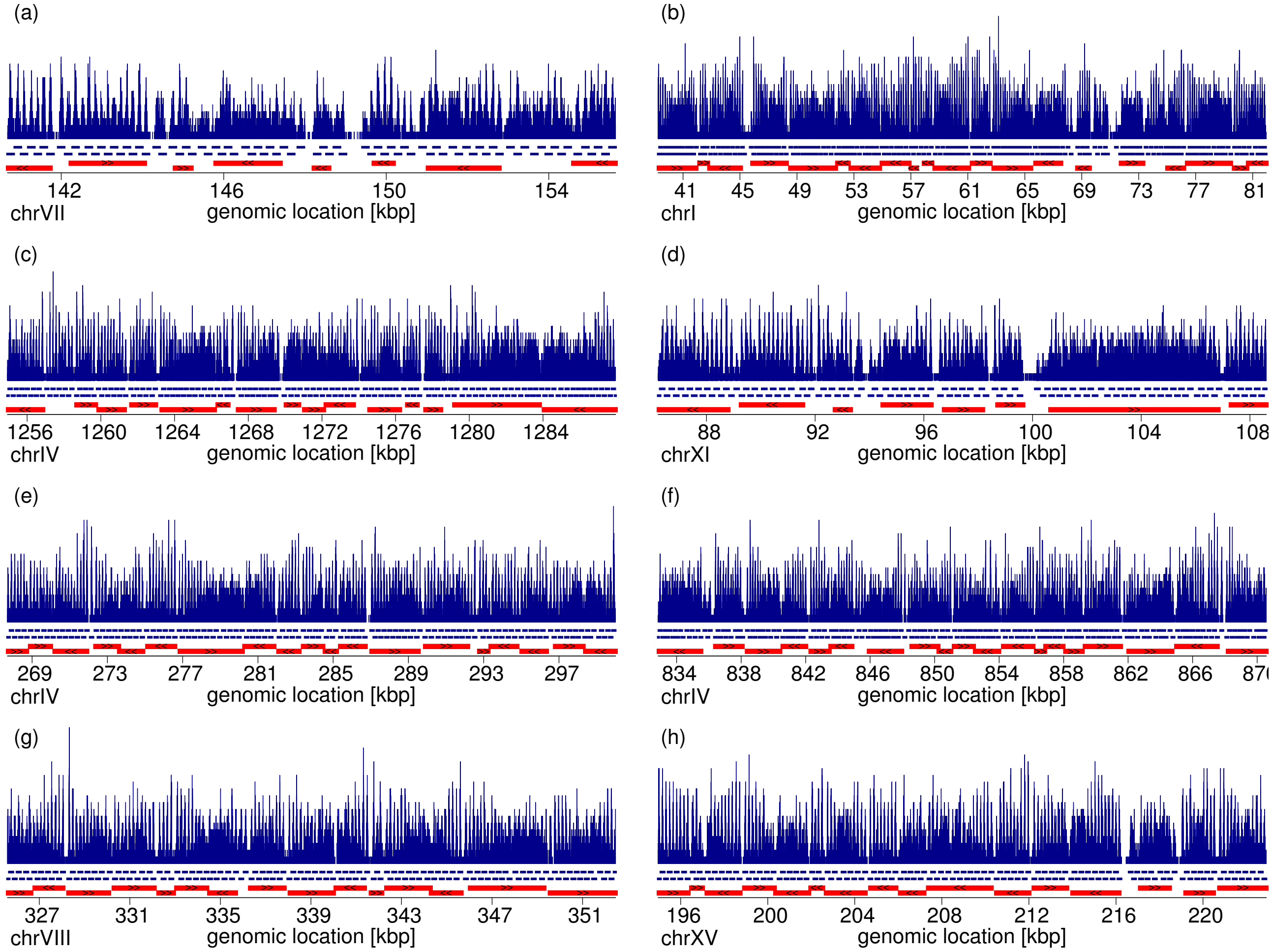

Fig. S1. Plots showing the data used as an input for simulations for each simulated region. Top: pile-up of reads from yeast MNase data (from Ref. (1)) for the indicated genomic region. Blue lines under the pile-ups show the positions of nucleosomes as found using the NucPosSimulator software (2); to aid visualisation these are shown in two rows with alternating nucleosomes on the top/bottom row. Red bars show genes (SacCer3 genome build).

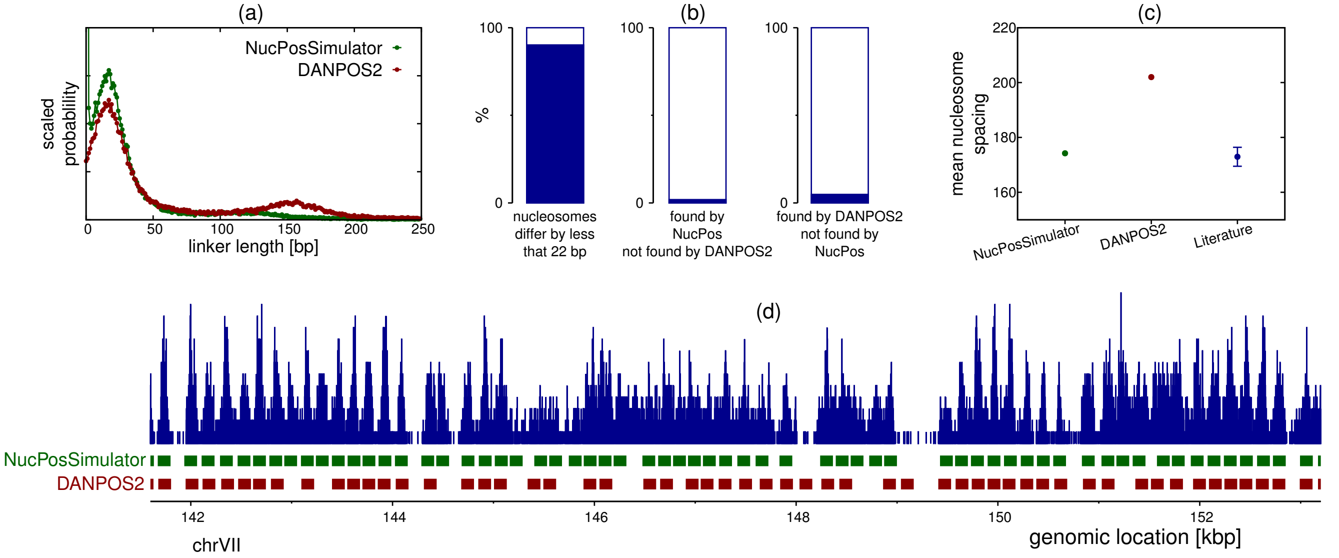

Fig. S2. Comparing methods for extracting nucleosome positions from MNase-seq data. (a) Plot showing the genome-wide distribution of DNA linker lengths for nucleosome positions obtained from data using NucPosSimulator (2) (green) and DANPOS2 (3) (red) using MNase-seq data from Ref. (1). **(b)** Bar plots summarising differences between nucleosome positions between the two software tools. Values are expressed as percentages of the total number of nucleosomes identified by the software. **(c)** Plot showing the mean nucleosome centre-to-centre spacing as found by each software tool compared to values quoted in the literature. Nucleosome free regions larger than 250 bp were assumed to be artefactual and so were not included in the mean. The value for the literature is obtained by averaging over nucleosome spacings for *Saccharomyces cerevisiae* quoted in Refs. (4–6), and the error bar shows the error in the mean (Ref. (6) quotes a value obtained in four previous studies). **(d)** Example nucleosome positions shown for a region on chrVIII. The raw pile-up from the MNase-seq data is shown (blue) above the nucleosome positions obtained from each software tool.

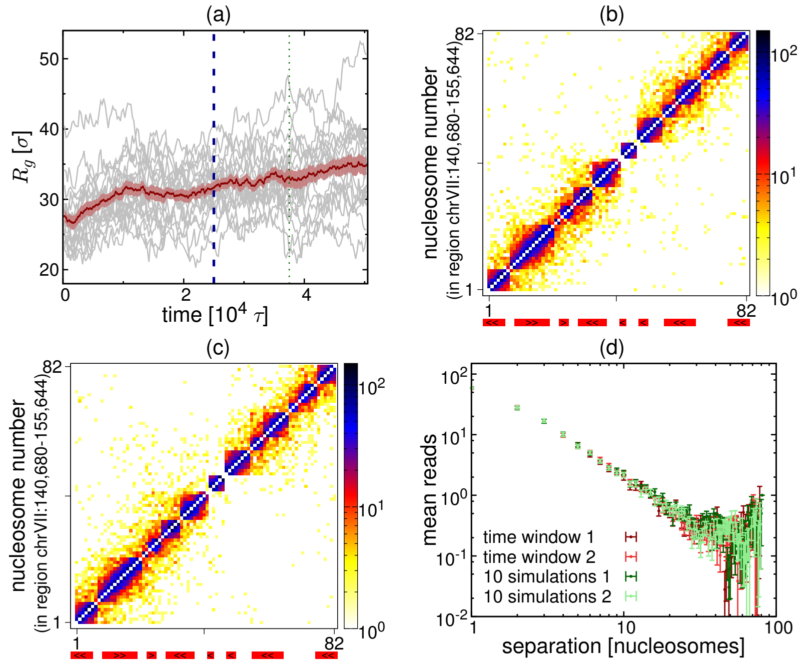

Fig. S3. The set of simulated chromatin configurations represent a sufficient sample of equilibrium conformations. (a) Plot from simulations of the region chrVII:140680-155644 showing how the radius of gyration R_g varies with time. Grey lines represent 20 independent simulations, and the dark red line shows the mean. The shaded red region represents the error in the mean. The region to the right of the blue dashed line represents the part of the simulation from which configurations are taken to generate contact maps. Units σ and τ are simulation length and time units representing 2.5 nm and 80 μs respectively, as detailed in *SI Appendix*. **(b)** Contact map obtained from 20 independent simulations, where the upper triangle is generated from configurations at times between the blue dashed and green dotted lines in (a), and the lower triangle is from configurations at times to the right of the green dotted line in (a). **(c)** Contact map obtained where the upper triangle is generated from configurations from 10 independent simulations, and the lower triangle is generated from configurations from 10 different independent simulations. **(d)** Plot showing mean reads *vs.* genomic separation found from each of the four contact maps show in (b) and (c). That is to say, plots are from the chrVII:140680-155644 region with two sets of configurations obtained from the time windows shown in (a) and two sets of 10 different independent simulations.

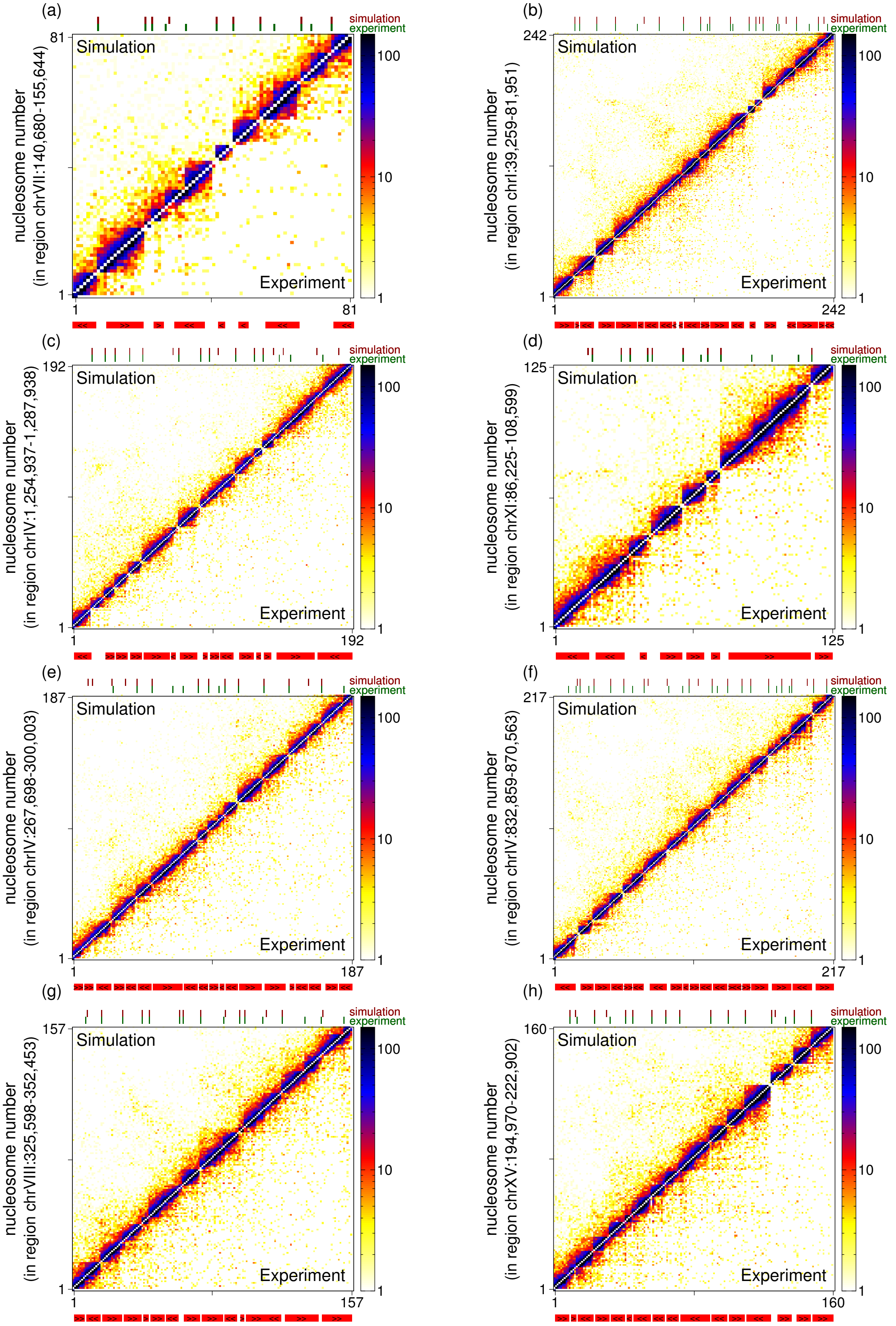

Fig. S4. Maps showing interactions between nucleosomes in each simulated region. The colour of the square at coordinates i, j indicates the number of MicroC reads corresponding to interactions between nucleosomes i and j (nucleosomes counted from 1 within each region). The lower triangle shows MicroC data from Ref. (7), while the upper triangle shows simulation results. Numbers on the axes show genomic position, but these are approximate, since nucleosome spacing is not regular. The locations of genes (mapped to the nearest nucleosomes) are shown below the plot (red bars; gene orientation is indicated with black arrowheads). Domain boundaries were called from each map (see *SI Appendix* for details), and these are indicated with ticks above the plot (upper row shows simulation boundaries in red, lower row shows experimental boundaries in green).

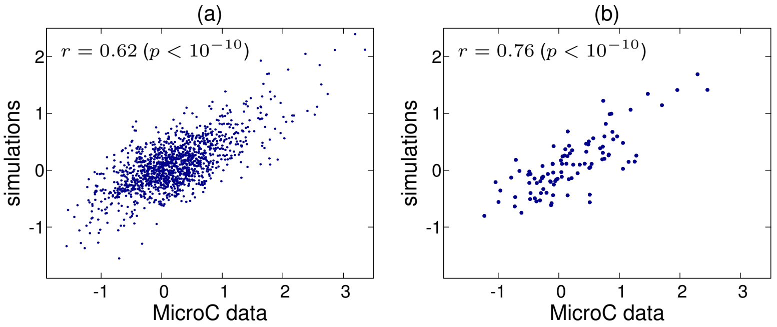

Fig. S5. Insulation score comparison between MicroC data and simulations (a) Scatter plot showing the nucleosome insulation score at all nucleosomes within the 8 simulated regions, for the MicroC data (8) and the simulations. The Spearman rank correlation between the two is indicated. **(b)** A similar plot is shown but only including insulation scores at the boundaries (those correctly predicted by simulations). Again the Spearman rank correlation between the two is indicated.

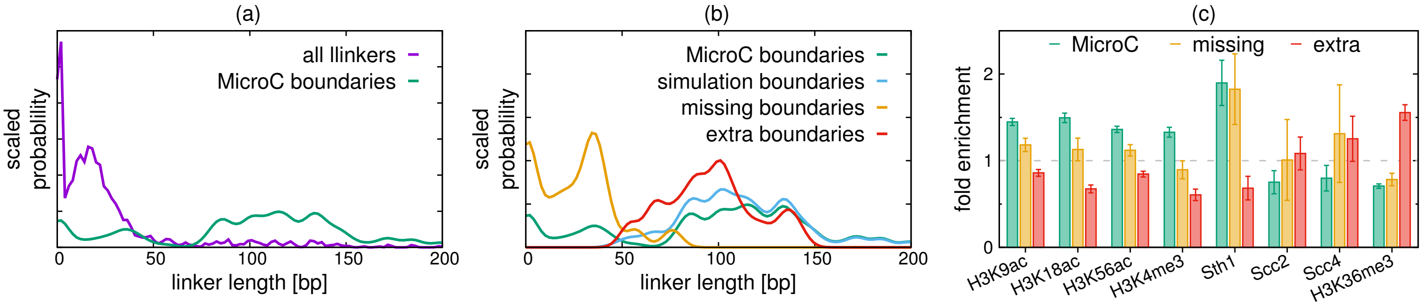

Fig. S6. Plots showing the properties of domain boundaries within the simulated regions. (a) The distribution of linker lengths across the simulated regions (purple) is plotted along side the distribution for linkers at boundaries called from MicroC data (green). A kernel density estimation method with bandwidth of 5 bp is is used, where curves are normalised to enclose unit area. **(b)** Similar distributions are shown for the boundaries found in simulations (blue), the "missing" boundaries (yellow; found in MicroC data but not predicted by simulations) and the "extra" boundaries (red; found in simulation but not present in MicroC data). Again curves are normalised to enclose unit area. **(c)** Mean fold enrichment of different histone modifications or protein binding levels are shown for the different classes of boundary. ChIP-seq data are obtained from Ref. (9) for histone modifications, and from Ref. (10) for protein binding. For histone marks fold-enrichment is against the ChIP input signal, whereas for proteins the fold-enrichment against the mean protein level across the simulated regions.

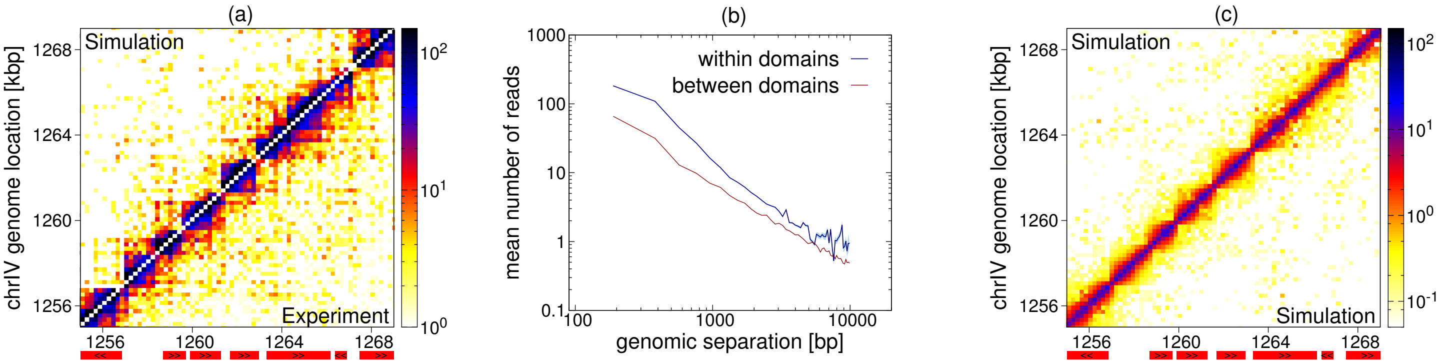

Fig. S7. Chromatin interaction maps which take into account variation of nucleosome occupancy. (a) The MicroC method only returns interaction information from nucleosomes. Here interactions are sorted into bins, and the number of reads for each pair of bins is scaled by the number of nucleosomes (as obtained using NucPosSimulator) overlapping the bins. This normalizes for the effect of variation of nucleosome occupancy (and NDRs). We use a bin size of 190 bp, as small as possible while having at least one (and up to 3) nucleosomes overlapping each bin. MicroC data and simulations are treated in the same way. Domains are still apparent, indicating that these are a real feature of yeast chromatin and not an artefact of the method. **(b)** Binned interaction maps such as those in (a) can be used to calculate the mean number or MicroC reads for interactions spanning a given genomic separation. Here we plot this, counting interactions between loci within the same domain and interactions between loci in different domains separately. MicroC data from all of the regions shown in Fig. S4 are included. There is a clear enrichment of interactions within the same domain – again this highlights that domains are a real feature of yeast chromatin and not an artefact of the method. **(c)** An alternative method to generate a contact map from simulations is to allow interactions from any pair of coarse-graining beads (see *SI Appendix* for details). Interactions are organized into bins (again 190 bp in width). Since bins can contain a mixture of DNA and nucleosome beads, they can have different numbers of beads – interactions are therefore normalized by the number of beads per bin. Again domains are apparent.

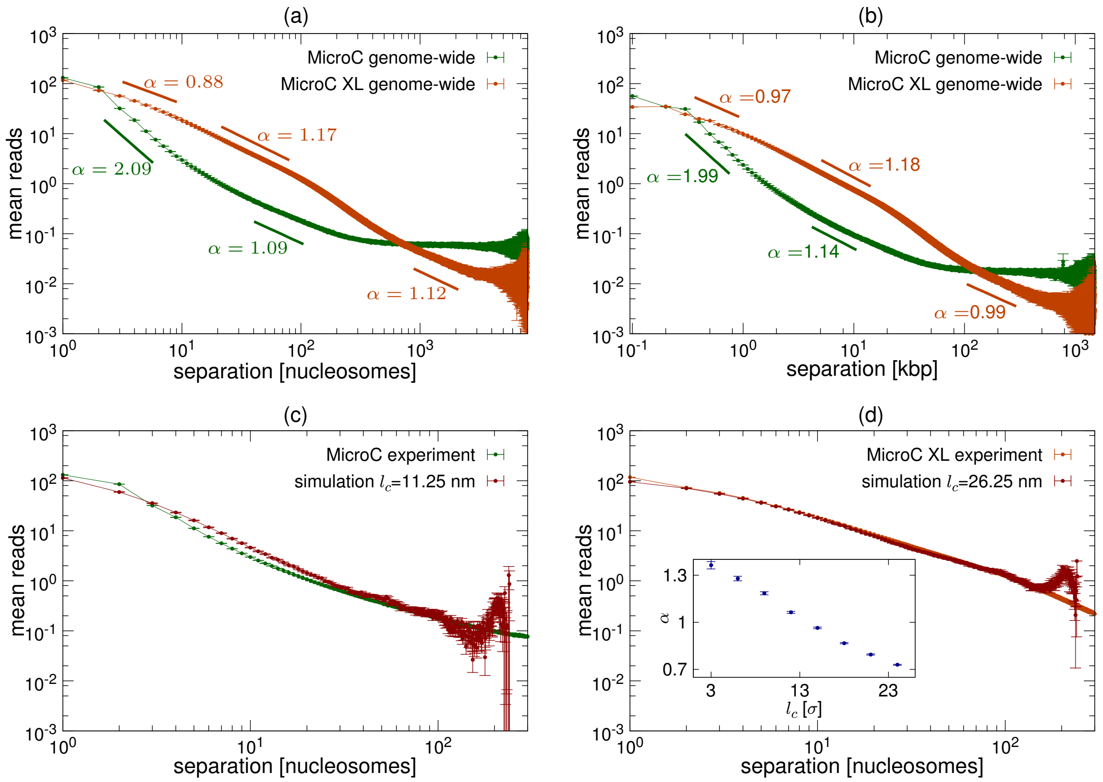

Fig. S8. Plots showing how, on average, the number of interaction reads between nucleosomes scales with their genomic separation. A linear relationship on a log-log plot implies power law behaviour (reads $\sim s^{-\alpha}$) and exponents α are approximated using linear fits to different ranges of the log-data. **(a)** Genome wide data from MicroC (green; obtained from Ref. (8)) and MicroC XL (orange; obtained from Ref. (7)) experiments are shown on the same plot. Separations are measured in nucleosomes, so a value of 1 means adjacent nucleosomes. **(b)** A similar plot is shown but here separations are measured in kbp. **(c-d)** Experimental data are shown alongside simulation results. Generating interaction maps from the simulated configurations depends on a "crosslinker length" parameter l_c, and a different values is required to give a reads *vs.* separation curve which fits to the different data sets, as indicated. The inset in (d) shows how the exponent from the $s = 10$-100 nucleosomes range varies as a function of l_c.

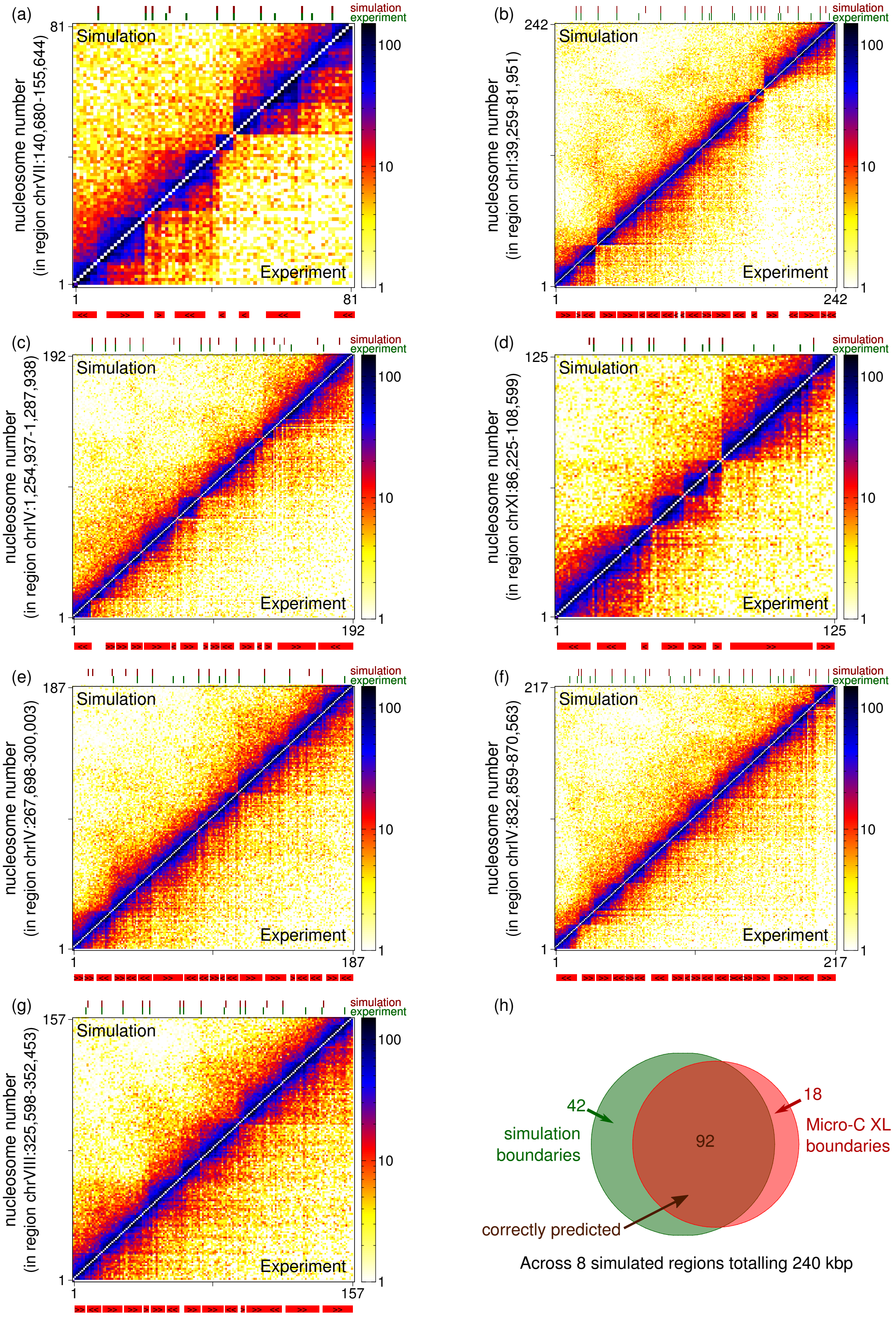

Fig. S9. Maps showing interactions between nucleosomes in each simulated region compared with MicroC XL data Plots showing interaction maps for 7 out of 8 simulated regions as in Fig. S4, but here the experimental data are those from MicroC XL experiments (7). Maps from the 8th simulated region are shown in Fig. 3 in the main text. Simulation maps are generated using a crosslinker length scale of l_c =26.25 nm. The bottom right plot shown a Venn diagram for boundaries identified across all simulated regions (the numbers are similar to those found for the comparison with the MicroC data shown in Fig. 2 in the main text).

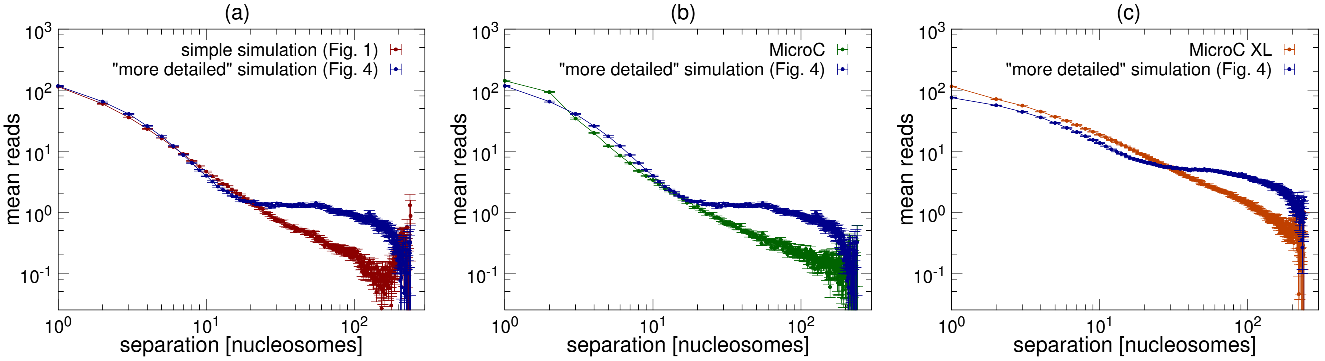

Fig. S10. Plots showing how the number of interaction reads between nucleosomes scales with their genomic separation for the more detailed model of Fig. 4. (a) Mean reads *vs.* separation for the two different simulation models. **(b)** Similar plot comparing the more detailed simulation model with MicroC data (8). Here a crosslinker length of $l_c = 4.5\sigma$ is used to generate the simulated map; changing this value does not lead to a curve which fits better to the data. **(c)** Similar plot comparing the more detailed simulation model with MicroC XL data (7). A crosslinker length of $l_c = 10.5\sigma$ is used to generate the simulated map; again, changing this value does not lead to a curve which fits better to the data.

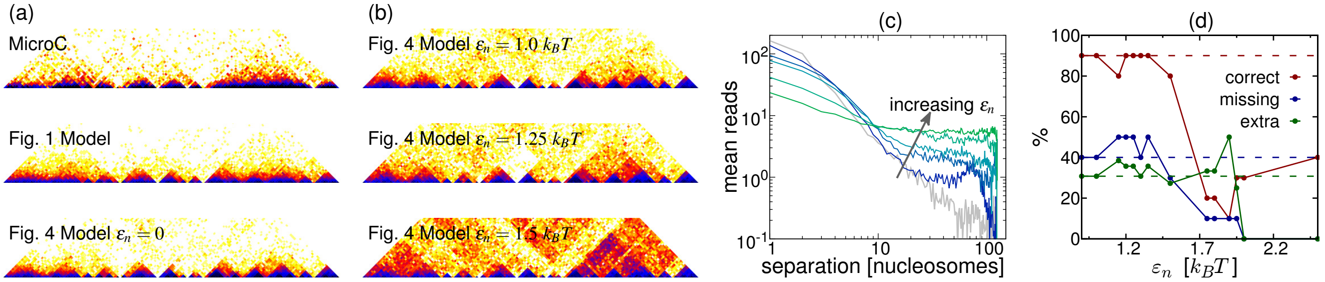

Fig. S11. A more detailed model with nucleosome-nucleosome attractive interactions. (a) Plot showing interaction maps (rotated through 45° and cropped) for the region chrXI:86225-108599 for MicroC data (top), simulations from the simple model described in Fig. 1 (middle), and simulations from the more detailed model described in Fig. 4 (bottom). **(b)** Similar plots but for a model where a short-ranged attractive interaction between nucleosomes is included. The three plots show results for different values of the attraction energy ϵ_n. **(c)** Plot showing mean reads *vs.* genomic separation for different values of the nucleosome attraction energy ϵ_n increasing as indicated (coloured lines; values ϵ_n=0.0, 0.9, 1.2, 1.5, and 1.8 k_BT). Grey line shows the plot from the MicroC data for this region. **(d)** Plot showing the proportion of called boundaries which are correct, missing or extra. Correct and missing boundaries are given as a percentage of the number of boundaries found in the MicroC data; extra boundaries are given as a percentage of the number of boundaries found in the simulation. Dashed lines indicate the value found in the simple model described in Fig. 1.

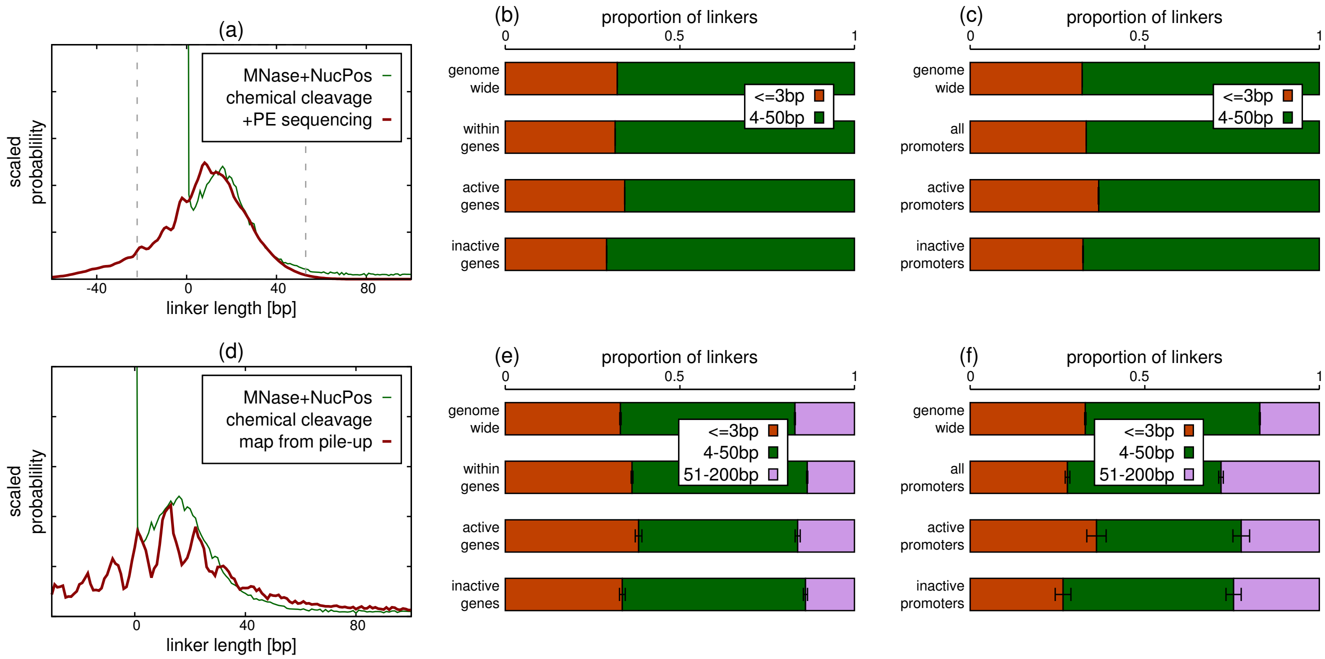

Fig. S12. Plots showing linker lengths obtained from chemical cleavage data from Ref. (11). (a-c) Plots of linker lengths obtained directly from chemical cleavage data using paired-end sequencing (see *SI Appendix* section10). The genome wide distribution of linker lengths (thick red line) is shown in (a), alongside the distribution obtained from MNase data using the NucPosSimulator software (thin green line; as shown in Fig. 5). Grey dashed lines enclose the range of linker lengths which are enriched by the experimental method. Linkers can be grouped into two size ranges, < 3 bp and 4-50 bp (with negative linkers referring to overlapping nucleosomes; this method biases against longer linkers), and by genomic location (within genes, or within the 500 bp upstream of genes) as discussed in the main text with reference to Fig. 5. Proportions of linkers falling within each group are shown in (b-c). Error bars are not shown as these are narrower than the lines. **(d-f)** Similar plots are shown for linker lengths based on a nucleosome positioning map obtained from pile-ups of cleavage sites. Here we use the map of "unique nucleosomes" (which forbids nucleosomes overlapping by more than 40 bp) which was provided as Supplementary Material in Ref. (11). The strong ~ 10.5 bp periodicity identified in that reference is clearly visible. In (e-f) three linker size ranges are shown: < 3 bp, 4-50 bp, and 51-200 bp. The error in the proportions for the < 3 bp and 51-200 bp cases are shown as error bars; non-overlapping error bars imply that the difference is statistically significant. The same trends as in the MNase based map are observed.

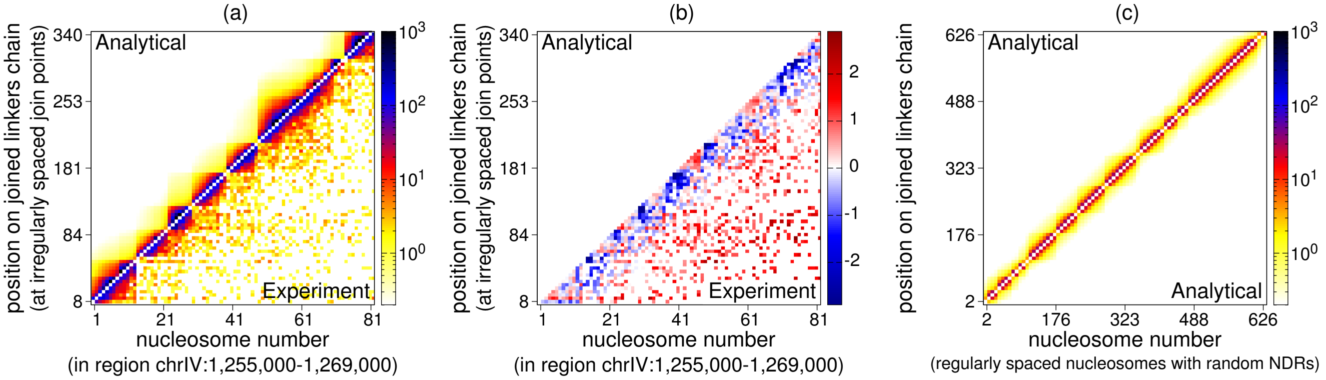

Fig. S13. Domains can be predicted analytically from DNA linkers. (a) From the region chrIV:1,255,000-1,269,000 we consider a bead-chain polymer obtained by replacing all nucleosome beads with a DNA bead to generate a uniform chain. Then, rather than generating a full contact map, we take only interactions between the beads which were originally nucleosomes. Assuming that the interaction probability for beads i and $i+s$ scales as s^{-1} we can generate an interaction map analytically (we assume reads $= 1000s^{-1}$ for $s > 0$). The result is a domain pattern (top left) similar to that obtained experimentally (bottom right). **(b)** A map of $\log(\text{observed}/\text{expected})$ interactions is shown. Since in the analytical case the map is generated simply as the expected values, this shows a uniform featureless map. For the experimental data the expected values are obtained by finding the average (over all simulated regions) interaction strength for the given *genomic* separation. Though this map is noisy, domains are still visible: boundaries lead to a dark-blue region close to the diagonal, while domain interiors have higher $\log(\text{observed}/\text{expected})$ values. **(c)** Interaction maps can be generated analytically for different hypothetical nucleosome spacings. Here nucleosome depleted regions of length 100 bp are positioned at random in an otherwise regular array of nucleosomes with 30 bp linkers. The domain pattern is very weak, showing that the full experimental distribution of linker lengths is important for determining the boundaries and their strength.

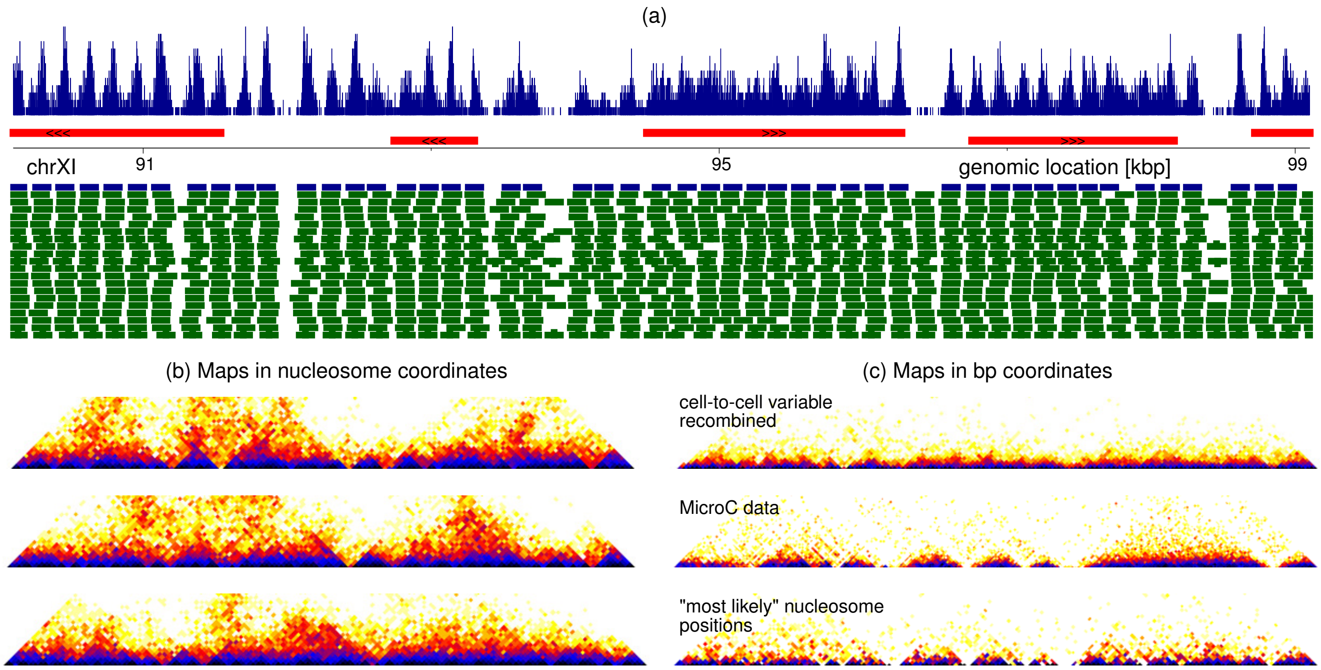

Fig. S14. Cell-to-cell variability in nucleosome positions: high variability. (a) Plot showing MNase-seq read pile-up for a region of chrXI with gene positions indicated by red bars (top). Nucleosome positions are show below, with the "most likely" positions shown as blue bars, and 20 different configurations shown as green bars (each row represents a different realisation of the nucleosome positions obtained from the data using NucPosSimulator with parameters which result in a high level of variation between configurations, as detailed in the text). Only part of the simulated region is shown. **(b)** Nucleosome interaction maps are shown from three different simulations of the chrXI:86225:108599 region where each used a different set of nucleosome positions. Nucleosome coordinates are used, and each simulation could have a different total number of nucleosomes, so horizontal positions on the maps are not aligned. **(c)** Interaction maps shown in bp coordinates for: (top) a set of 20 simulations each using a different set of nucleosome positions as shown by green bars in (a); (middle) MicroC data from Ref. (8); and (bottom) a set of 20 simulations each using the same set of "most likely" nucleosome positions as shown as blue bars in (a).

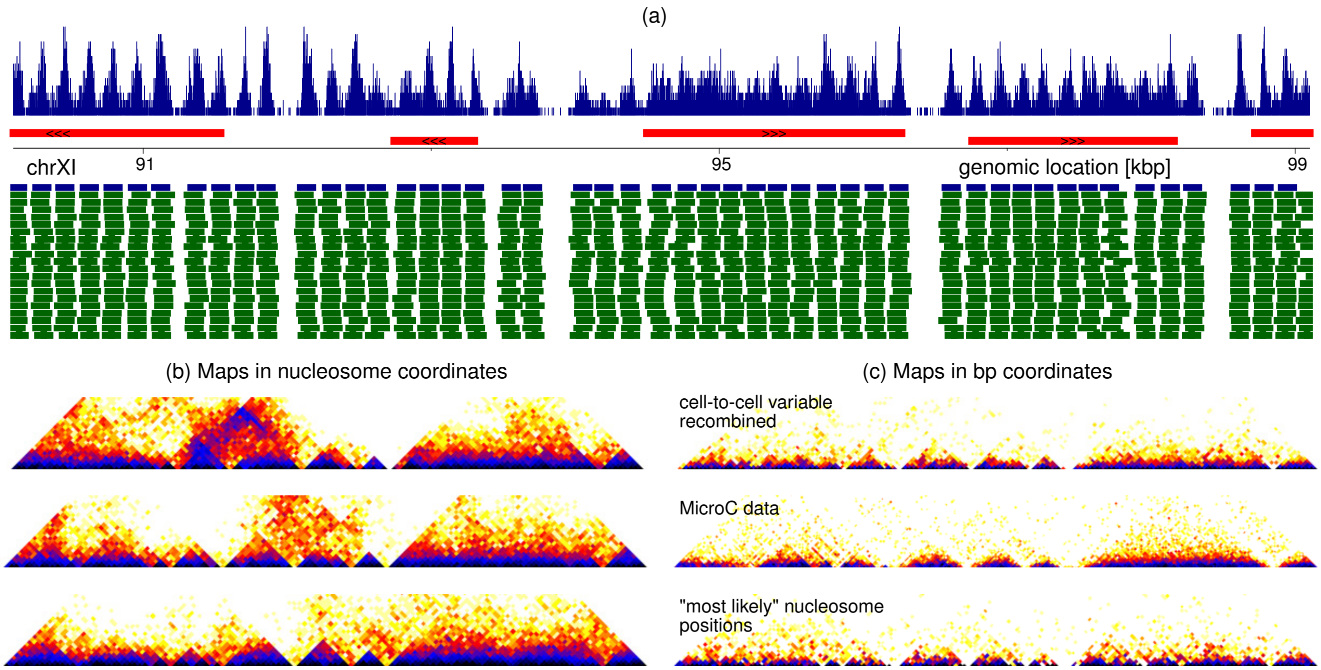

Fig. S15. Cell-to-cell variability in nucleosome positions: low variability. (a) Plot showing MNase-seq read pile-up for a region of chrXI with gene positions indicated by red bars (top). Nucleosome positions are show below, with the "most likely" positions shown as blue bars, and 20 different configurations shown as green bars (each row represents a different realisation of the nucleosome positions obtained from the data using NucPosSimulator with parameters which result in a low level of variation between configurations, as detailed in the text). Only part of the simulated region is shown. **(b)** Nucleosome interaction maps are shown from three different simulations of the chrXI:86225:108599 region where each used a different set of nucleosome positions. Nucleosome coordinates are used, and each simulation could have a different total number of nucleosomes, so horizontal positions on the maps are not aligned. **(c)** Interaction maps shown in bp coordinates for: (top) a set of 20 simulations each using a different set of nucleosome positions as shown by green bars in (a); (middle) MicroC data from Ref. (8); and (bottom) a set of 20 simulations each using the same set of "most likely" nucleosome positions as shown as blue bars in (a).

Supporting Information Text

1. Chromatin model

A. DNA model. Following previous work (12–14) we model linker DNA as a bead-and-spring polymer, where beads of diameter 2.5 nm represent 7.35 bp of DNA. The ith bead in the chain, having position $\mathbf{r}_i$ is connected to the $i+1$th bead with a finitely extensible non-linear elastic (FENE) spring: the associated potential is given by

$$U_{\rm FENE}(r_{i,i+1}) = U_{\rm WCA}(r_{i,i+1}) - \frac{K_{\rm FENE}R_0^2}{2}\log\left[1-\left(\frac{r_{i,i+1}}{R_0}\right)^2\right], \quad [1]$$

where $r_{i,i+1} = |\mathbf{r}_i - \mathbf{r}_{i+1}|$ is the separation of the beads, and the first term is the Weeks-Chandler-Andersen (WCA) potential

$$\frac{U_{\rm WCA}(r_{ij})}{k_BT} = \begin{cases} 4\left[\left(\frac{d_{ij}}{r_{ij}}\right)^{12} - \left(\frac{d_{ij}}{r_{ij}}\right)^{6}\right]+1, & r_{ij} < 2^{1/6}d_{ij} \\ 0, & \text{otherwise,} \end{cases} \quad [2]$$

which represents a steric interaction preventing adjacent beads from overlapping. In Eq. (2) d_{ij} is the mean of the diameters of beads i and j. The diameter of the DNA beads is a natural length scale with which to parametrize the system; we denote this by σ, and use this to measure all other length scales. The second term in Eq. (1) gives the maximum extension of the bond, R_0; throughout this work we use $R_0 = 1.6\ \sigma$, and set the bond energy $K_{\rm FENE} = 30\ k_BT$ for linker DNA beads.

The bending rigidity of the polymer is introduced via a Kratky-Porod potential for every three adjacent DNA beads

$$U_{\rm BEND}(\theta) = K_{\rm BEND}\left[1-\cos(\theta)\right], \quad [3]$$

where θ is the angle between the three beads as given by

$$\cos(\theta) = [\mathbf{r}_i - \mathbf{r}_{i-1}]\cdot[\mathbf{r}_{i+1}-\mathbf{r}_i], \quad [4]$$

and $K_{\rm BEND}$ is the bending energy. The persistence length in units of σ is given by $l_p = K_{\rm BEND}/k_BT$.

Finally, steric interactions between non-adjacent DNA beads are also given by the WCA potential [Eq. (2)].

B. Nucleosome model of Figure 1. In our first simple model, as depicted in Fig. 1a, nucleosomes are represented by 10 nm (4 σ) diameter beads, connected to linker DNA beads using FENE bonds according to Eq. (1) but with the appropriate choice for R_0 (i.e. $R_0 = 3.6\ \sigma$ for the bond between a DNA bead and a nucleosome bead, or $R_0 = 5.6\ \sigma$ for the bond between two nucleosome beads); as before steric interactions between nucleosome beads, and between nucleosome and DNA beads are given by the WCA potential, with d_{ij} being the mean of the diameters of the two beads.

C. More detailed geometry nucleosome model of Figure 4. In the more detailed model, as depicted in Fig. 4a, nucleosomes are represented by a rigid body composed of five component beads including four 5 nm (2 σ) beads and a 2.5 nm (σ) connector bead (see Fig. 4a). The four core beads are arranged with their centres on the corners of a square of size 4.2 nm (1.68 σ); the connector bead is positioned 5.75 nm (2.3 σ) from the centre of the square. Linker DNA beads are connected to nucleosome connector beads using harmonic springs with the associated potential

$$U_{\rm HARM}(r_{i,i+1}) = K_{\rm HARM}(r_{i,i+1}-R_0)^2, \quad [5]$$

where $r_{i,i+1} = |\mathbf{r}_i - \mathbf{r}_{i+1}|$ is the separation of the beads, and $R_0 = 1.1\ \sigma$ is the equilibrium separation.

To constrain the entry-exit angle for linkers emerging from a nucleosome, a bending interaction between three connected DNA-connector-DNA beads is given by

$$U_{\rm NUC-BEND}(\theta) = K_{\rm BEND}\left[1-\cos(\theta-\theta_0)\right], \quad [6]$$

where θ is the angle between the three beads, and θ_0 is the desired equilibrium angle, set at $\theta_0 = 72°$, so as to match the entry-exit angle measured from the canonical nucleosome crystal structure (15). We set the interaction energy to be the same as that used for the linker DNA beads.

2. Simulation Method

In our coarse grained molecular dynamics simulations, the position of the ith bead $\mathbf{r}_i$ changes in time according to the Langevin equation

$$m_i\frac{d^2\mathbf{r}_i}{dt^2} = -\nabla U_i - \gamma_i\frac{d\mathbf{r}_i}{dt} + \sqrt{2k_BT\gamma_i}\boldsymbol{\eta}_i(t), \quad [7]$$

where m_i is the mass of bead i, γ_i is the friction it feels due to an implicit aqueous solvent, while $\boldsymbol{\eta}_i$ is a vector representing random uncorrelated noise which obeys the following relations

$$\langle\eta_\alpha(t)\rangle = 0 \quad \text{and} \quad \langle\eta_\alpha(t)\eta_\beta(t')\rangle = \delta_{\alpha\beta}\delta(t-t'). \quad [8]$$

The noise variance is scaled by the thermal energy, given by the Boltzmann factor k_B multiplied by the temperature of the system T, taken to be 310 K for a cell. The potential U_i is a sum of interactions between bead i and all other beads, as described above. For simplicity we assume that all beads in the system have the same mass and friction $m_i \equiv m$, and $\gamma_i \equiv \gamma$. Eq. (7) is solved using the LAMMPS software (16) which uses a standard velocity-Verlet algorithm; we use a time step of $\Delta t = 0.005\ \tau$ (where τ is the simulation time unit, as defined in the next section).

Our simulations are initialized such that the nucleosome and DNA bead positions follow a random walk; we then evolve the dynamics for 122 τ to obtain an equilibrium polymer conformation. We simulated for a further $50\times10^3\ \tau$ and saved configurations every 250 τ; this was repeated 20 times for each region using a different set of random numbers to generate the noise $\boldsymbol{\eta}_i(t)$ each time. We use periodic boundary conditions, with a simulation box size of $400\times400\times400\ \sigma$, meaning that the system is dilute (though, as detailed in the next section, the diffusivity of the chromatin is mapped to *in vivo* measurements meaning this effectively takes into account slow-down of the dynamics due to macromolecular crowding).

3. Mapping simulation units to physical units

As detailed above our simulations use length units of σ=2.5 nm, and energy units of k_BT; masses are given in units of the mass of a DNA bead, approximately 8×10^{-24} kg. A choice of $K_{\rm BEND} = 20\ k_BT$ therefore gives a realistic DNA persistence length of $l_p = 20\ \sigma = 50$ nm.

To map between simulation and real time units, we first note that the above defined length, energy and mass units lead

to a natural time unit $\tau = \sqrt{m\sigma^2/k_BT}$. Another important time scale is the Brownian time $\tau_{\rm B} = \sigma^2/D_i$, which is the time scale over which a DNA bead diffuses across its own diameter σ. Here D_i is the diffusion constant for bead i, given through the Einstein relation by $D_i = k_BT/\gamma_i$. With our choice of $\gamma_i = 1$ this means $\tau_{\rm B} = \tau$. To map to real times we measure the mean squared displacement (MSD) for all beads; we then find the value of $\tau_{\rm B}$ which gives the best fit to experimental results from Ref. (17), who measured the MSD for various chromatin loci in live yeast cells. This results in $\tau_{\rm B} = 80$ μs, meaning that each 10^7 time step simulation run represents approximately 4 s of real time.

4. Analysis of MNase data

A number of methods have been developed to obtain nucleosome positioning information *in vivo*, and the most extensively employed is micrococcal nuclease (MNase) digestion combined with high-resolution microarrays or high-throughput sequencing. The premise is that MNase will digest any DNA which is not protected by its association with proteins, and that mapping of undigested fragments reveals which DNA regions were associated with nucleosomes. For most of this work we used MNase-seq data, but in section 10 below we consider some alternative methods.

We used MNase-seq data published in Ref. (1) (available at GEO:GSM53721). Paired-end reads were aligned to the *Saccharomyces cerevisiae* reference genome (SacCer3 build) using bowtie2 (18); duplicates and any reads with mapping quality less than 30 were removed. Nucleosome coverage maps (e.g. blue bar plots in Fig. 1c and Fig. S1) were then obtained by piling-up the centre points of each paired-end read.

Since MNase-seq data is obtained from a population of cells, and it is expected that nucleosomes positioning will show cell-to-cell variability, obtaining an average or "most likely" set of nucleosome positions requires a statistical approach (and we note that this is a distinct problem from understanding the factors which drive nucleosome positioning *in vivo*, e.g. DNA sequence and chromatin remodelling factors (19)). A number of methods for obtaining such positions have been presented in the literature, and these tend to fall into two categories: methods which infer an effective nucleosome binding energy landscape from the data, and methods which find peaks from the pile-up of fragments. The NucPosSimulator software (2) is a popular tool which falls into the first category. In short, it takes the centre point of each paired-end read and builds a frequency count profile, which is then used to generate an effective potential landscape for nucleosome binding. A Metropolis Monte-Carlo algorithm is then used to add, remove and move nucleosomes within this landscape. We use NucPosSimulator in "simulated annealing" mode, where the system starts at a high temperature (where nucleosomes will be highly dynamic) before being slowly cooled. During this process the nucleosomes settle into their most likely positions. Full details of the software are given in Ref. (2).

A popular tool which instead uses peak finding to extract nucleosome positions from MNase data is DANPOS2 (3). Comparing the output generated by DANPOS2 and NucPosSimulator we found that over 90% of nucleosome positions changed by less than 22 bp between the two maps, but NucPosSimulator tended to identify more nucleosomes than DANPOS2 (4.9% of nucleosomes found by NucPosSimulator were not found by DANPOS2, and 1.9% of nucleosomes found by DANPOS2 were not found by NucPosSimulator). These results are summarized in Fig. S2. This indicates that there is only a small difference in nucleosomes identified by these two software tools; DANPOS2 tended to find more nucleosome free regions than NucPosSimulator, leading to a slightly larger mean nucleosome spacing. We decided to use the positions generated by NucPosSimulator in our simulations because the average nucleosome spacing it generated was closer to that quoted in the literature, and there was a better visual comparison between the raw MNase pile-up and the extracted positions (Fig. S2d).

5. Analysis of MicroC and MicroC XL data

MicroC data are obtained from Ref. (8) (available at GEO: GSE68016), while MicroC XL data are obtained from Ref. (7) (available at GEO:GSE85220, specifically we used samples GSM2262329, GSM2262330 and GSM2262331). We follow the same processing procedure for each data set, and this is similar to that described in Ref. (8). After trimming adapters from the paired-end data we use bowtie2 (18) to align reads to the *Saccharomyces cerevisiae* reference genome (SacCer3 build); the data were treated as single-end reads, since ligation fragments will not form proper pairs when aligned. Duplicates were removed, and reads filtered to retain those where both pairs attained a mapping quality of at least 30. Following Ref. (8) we further filter reads according to the strand each read in the pair maps to, in order to avoid including reads resulting from runs of undigested nucleosomes. Interactions can then either be binned to obtain standard interaction maps, or can be mapped onto specific nucleosomes to obtain a nucleosome-nucleosome interaction map.

To generate nucleosome level interaction maps we use the nucleosome positions generated by NucPosSimulator from the MNase-seq data as detailed above. Treating each of the pair from each MicroC read separately, reads which overlap with a single nucleosome are unambiguous; reads which do not overlap with a nucleosome, but map to a position where their centre point is within 200 bp of the centre of one or more nucleosomes are assigned to their closest nucleosome. Reads which overlap with more than one nucleosome are assigned to that with which they have the largest overlap. Reads which do not map within 200 bp of a nucleosome, or which overlap with two nucleosomes by the same amount are discarded. Only read pairs where both members of the pair are assigned to nucleosomes are retained as informative interactions. For the MicroC data, across 20 replicates, starting with 73,943,603 interactions, we were able to assign 73,803,602 of these unambiguously to pairs of nucleosomes; i.e. less than 1% of read pairs were discarded as it was ambiguous as to which nucleosomes they represented. For the MicroC XL data, across 3 replicates, starting with 130,958,525 interactions, we were able to assign 114,652,179 of these unambiguously to pairs of nucleosomes, this time discarding less then 0.05% of read pairs.

6. Generating Nucleosome interaction maps from simulations

From our simulations we obtain the positions of all beads in 2000 independent configurations for each region as detailed above. In a MicroC experiment nucleosomes are cross-linked, unprotected DNA is digested, and then protected DNA fragments are ligated: we expect that the probability that DNA

from two nucleosomes are ligated together is a function of their 3-D separation. To mimic this process *in silico* we pick two nucleosomes at random from a simulated configuration, we then accept this as an interaction with a probability $P(r)$ which is a function of the nucleosomes separation r, and reject it otherwise. For simplicity we choose an exponential function $P(r) = e^{-r/l_c}$, with interaction (or "crosslinking") length scale l_c. For a simulation with N nucleosomes, we perform this operation N^2 times, and then repeat this for each configuration. To obtain a number of simulated interactions which is similar to the number of MicroC reads for a given region we can repeat this entire process multiple times. Finally, to obtain a map which is comparable to the data, we scale all interaction counts by a factor γ which results in there being the same total number of reads in the simulated and experimental interaction maps. We find that the observed domain pattern is largely insensitive to the value of the parameter l_c, but this strongly affects the mean interactions *vs.* genomic separation (as detailed in the main text, and section 8 below). To compare with experiments we generated simulated interaction maps using different values of l_c in the range 3–30 σ, and then used the value which gave the best fit to the experimental interactions *vs.* separation plot (Fig. S8c-d; this was different for the MicroC and MicroC XL data, as detailed in the main text).

In Fig. S7b an alternative method is used to generate the map, where instead of picking two nucleosomes at random, any two beads (nucleosome or DNA) can be chosen. The rest of the procedure follows as above, but then the interactions are sorted into bins with a fixed width in bp. Since each bin can contain a mixture of DNA and nucleosome beads, they can contain different numbers of beads, so interactions are further normalized by the number of beads per bin.

To ensure that the contact maps are generated from a sample of configurations which are representative of equilibrium conformations, we tracked the radius of gyration, R_g, as a function of time (Fig. S3a), ensuring that we use configurations at a point after this had stopped systematically changing. This quantity is defined by

$$R_g^2 = \frac{1}{N} \sum_{i=1}^{N} \left(\mathbf{r}_i - \mathbf{r}_{\text{mean}} \right)^2 ,$$

where $\mathbf{r}_i$ is the position of the ith bead in the fibre, and $\mathbf{r}_{\text{mean}}$ is the mean over all bead positions. We further checked that the configurations were not changing in time by generating contact maps from two different time windows (Fig. S3b). There was no appreciable difference between the maps (at least close to the diagonal, where we make measurements to compare with data). We also checked that considering 20 independent simulations was enough to ensure a representative sample by generating contact maps from each of two sets of 10 independent simulations (Fig. S3c). Again there was no appreciable difference between the maps. Finally we compared mean reads *vs.* genomic separation plots for each of these contact maps, showing no appreciable difference within the standard error (Fig. S3d).

7. Calling Boundaries from Interaction Maps

To find domain boundaries for both MicroC data and the simulated interaction maps we use a "sliding box" algorithm. Essentially a square box is placed off the diagonal of the interaction map (with its corner on $i, i+1$), and we sum the values for nucleosome interactions falling with the box. We then slide the box along the diagonal, nucleosome-by-nucleosome to obtain a boundary signal as a function of box position. Symbolically the signal at nucleosome k (used to determine if there is a boundary between nucleosomes k and $k+1$) is given by

$$s_k = \frac{1}{2d} \sum_{i=k-d+1}^{k} \sum_{j=k+1}^{k+d} x_{ij} \quad \text{for } d < k < N-d,$$

where x_{ij} is the number of interactions between the ith and jth nucleosome, N is the number of nucleosomes in the region and we use $d = 10$. At the edges of a region (i.e. $k < d$ or $k > N - d$) we use the same function but reduce d, e.g. for $k = 4$ we set $d = 3$. This is essentially the same as the algorithm used in Ref. (8), where for each nucleosome the number of upstream to downstream interactions (within some range) is counted.

This signal gives a measure the number of interactions between regions either side of a given nucleosome (the lower the value the fewer crossing interactions), so minima in s_k are potential boundaries. To call a boundary at a minima we require that value of s_k is smaller than its local average by at least some threshold factor; i.e. we define

$$\bar{s}_k = \frac{1}{9} \sum_{i=k-4}^{k+4} s_i ,$$

and then if $s_k < \gamma \bar{s}_k$ we call the minima a boundary. We tune the value of the factor γ by visual inspection of the called boundaries and the interaction map; we choose this separately for the MicroC and MicroC XL data, but keep the same values for calling boundaries from the corresponding simulation maps. Specifically for MicroC data we set $\gamma = 0.85$ and for MicroC XL data $\gamma = 0.97$. We note that due to noise in the data, occasionally the boundaries found by the algorithm are not the same as would be expected from visual inspection of the interaction maps: this highlights the difficulty in unambiguously defining domains and boundaries within 3C based interaction maps. We find that the algorithm works better for the MicroC data than for the MicroC XL, though visually the same domains are clearly present in the contact maps for each data set.

We deem any boundary found in the simulated interaction map to be a "correct prediction" when it is within one nucleosome of the position of an experimental boundary. As detailed in the main text, we denote a boundary which is found in the simulations but not the MicroC data an "extra boundary", and any boundary found in the MicroC but not in the simulations is a "missing boundary". To obtain a p-value for the level of agreement between a set of simulated boundaries and the experimental data, we generated a set of random boundary positions which have the same statistics (i.e. the same mean boundary separation), counted the number of correct boundaries, and then repeated this many times. In this way we estimate the probability that the boundaries could be correctly positioned due to random chance (the probability that the null hypothesis is true).

To obtain an insulation signal at each nucleosome we scale s_k by its mean value across all regions and take the negative

log

$$u_k = -\log\left(\frac{s_k}{\langle s_k \rangle}\right),$$

so that the more interactions which are observed between regions either side of nucleosome k, the lower the insulation signal. In Fig. S5 we plot the u_k found from simulations *vs.* that found from the MicroC data. As a quantitative comparison we calculate the Spearman rank correlation coefficient, which would give the same value independent of the logarithm and scaling. The insulation signal at a domain boundary can be viewed as a measure of the strength of that boundary (Fig. S5b).

8. Interactions *versus* genomic separation: genome-wide analysis

In Fig. 3b in the main text we plot the mean number of MicroC (or MicroC XL) interactions as a function of genomic separation averaged over the regions which we simulated. As discussed there the plot is different for the two different experimental data sets, and by comparison with our simulations (Fig. 3d in the main text and also Fig. S8c-d) we found that the slope of this plot depends on the "crosslinker length". In Fig. S8a-b we show a similar plot of the genome-wide average for the two experimental data sets. Noting that linear regions on a log-log plot imply a power law relationship, we find that there are three different linear regimes. In Fig. S8a we observe that for the MicroC data the number of reads initially decreases steeply with genomic separation (with an exponent close to 2), but then this becomes shallower, and there is another linear regime with exponent close to 1. There is a plateau at large separations – as discussed in Ref. (8) the original MicroC method fails to capture long range interactions. The MicroC XL data shows a much shallower slope for small separation, with an exponent less than 1; it then becomes steeper with a linear regime with a slope close to 1. At long ranges (separation $s > 500$) there is again a slope close to 1. It is unclear what the origin of these different regimes is, though we note that HiC data typically shows an exponent close to 1 for large separations. Here we plot separations in units of nucleosomes, but similar exponents are observed if we instead plot separations as DNA length (Fig. S8b).

9. Further model refinements also fail to show improved agreement with data

Given the surprising result that including a more detailed nucleosome geometry in the model does not give an improved agreement with the data, we considered two further refinements to the model described in Fig. 4.

First we included a short ranged attractive interaction between nucleosomes to model direct nucleosome-nucleosome interactions which could be mediated by surface charges or histone tail interactions, see Fig. S11. To do this we included a Lennard-Jones interaction between any pair of nucleosomes, given by

$$U_{\mathrm{LJcut}}(r) = \begin{cases} U_{\mathrm{LJ}}(r) - U_{\mathrm{LJ}}(r_{\mathrm{cut}}), & r < r_{\mathrm{cut}} \\ 0, & \text{otherwise}, \end{cases} \quad [9]$$

where

$$U_{\mathrm{LJ}}(r) = 4\epsilon_n \left[\left(\frac{d_n}{r}\right)^{12} - \left(\frac{d_n}{r}\right)^{6} \right], \quad [10]$$

where r is the separation between the centre points of the nucleosomes (a point at the geometric centre of the four large beads making up the rigid body), ϵ_n is the interaction energy, $d_n = 2.0\,\sigma$, and $r_{\mathrm{cut}} = 3.0\,\sigma$ is the range of the interaction. We found no improvement in agreement with the data compared to the simple model (comparing the boundary calls, insulation signal, and reads *vs.* genomic separation plots, Fig. S11). Since in Ref. (8) the authors noted that nucleosomes within inactive genes tended to interact more, we reasoned that the presence or lack of certain histone modifications could be used to identify nucleosome which might interact attractively – however a model where the nucleosome-nucleosome interaction was present only in some regions of the simulated fibre also did not lead to an improvement in agreement with data.

Second, we considered that certain histone modification might lead to partial unwrapping of DNA from the histone octamer. Although our model is too simple to include this in detail, we reasoned that it could be included in a simple way by removing the angle constraint on the entry/exit DNA for a subset of nucleosomes with acetylation modifications. Again we saw no improvement in agreement with the MicroC data.

10. Genome-wide nucleosome spacing: further analysis

In the main text we present some analysis of nucleosome linker lengths based on MNase-seq data. We note here that similar results are obtained using data obtained from site-directed DNA cleavage experiments (from Ref. (11)), which offer higher resolution. Specifically the method uses an *Saccharomyces cerevisiae* mutant with a unique cysteine in histone H4, which allows chemical cleavage of DNA at precise locations near the nucleosome dyad.

Linker lengths can be obtained by two approaches. First, the fragments that the experiment yields are stretches of DNA between two nucleosome centres; these can be run on an agarose gel to reveal bands corresponding to DNA fragments from pairs, triplets etc. of nucleosomes. Purifying the lowest molecular weight band and performing paired end sequencing gives directly the separation of pairs of nucleosomes as they appear in single cells; however, this purification selects against longer linkers (fragments representing linker lengths between ~ -22 and 53 bp are enriched for; here a negative linker length means that the nucleosomes overlap). Fig. S12a shows the linker length distribution obtained in this way, alongside that from the MNase data and NucPosSimulator discussed in the main text. Two features of note are that there are negative length values, which indicates overlapping nucleosomes, and that peaks at ~ 10 bp intervals are visible; we discuss these points in detail below. Though we cannot use the chemical cleavage data to analyse long linkers, we can still count the number short (< 3 bp) and medium (4-50 bp) length linkers in different genomic regions. Fig. S12b-c shows that, consistent with the MNase based positioning (Fig. 5e-f), both within gene bodies and within the region upstream of their TSS, the proportion of linkers which are shorter than 3 bp is higher for active than for inactive genes. The fact that a similar distribution, and similar trends for different genomic regions, can be observed implies that the features discussed in the main text are not artefacts arising because nucleosome positions are based on a population level map.

The second approach to obtaining linker lengths from the

chemical cleavage data, as detailed in Ref. (11), is to pile-up the strand-dependent cleavage points across the genome to form a nucleosome position map. Due to the specific pattern of cleavage positions on opposite strands, nucleosome centres can be identified from the strand-specific shape of the peaks (since the method is sensitive to the shape of the peak and not the height, the purification bias discussed above does not have an effect (11)). Here we used the "unique nucleosome map" provided as supplementary material in Ref. (11); this does not allow nucleosomes to overlap by more than 40 bp (taking the positions with the highest score where larger overlaps occur). We note that, like MNase data, this map gives a "population" picture, and may not represent the situation within individual single cells. From this map we obtain the proportions of short ($<$ 3 bp), medium (4-50 bp), and long (51-200 bp) linkers as in Fig. 5, and again find that short linkers appear more frequently within active than inactive genes and promoter regions (Fig. S12e-f), and that long linkers (NDRs) appear more frequently in promoter regions than genome wide (though the difference between active and inactive promoters was not statistically significant, Fig. S12f).

As detailed in Ref. (11), the higher-resolution obtained by the chemical cleavage method reveals that a significant proportion of nucleosome overlap with the territory of their neighbours, and that there is a strong $\sim$ 10.5 bp periodicity in the linker length distribution. The former manifests itself in the data as nucleosome centre-centre DNA fragments shorter than 147 bp (negative linker lengths); this has been studied using an *in vitro* positioning system (20) – there the authors observed that partial unwrapping of DNA from the histone octamer allows nucleosomes to invade their neighbour's territory and that extreme overlapping coincides with a loss of an H2A-H2B dimer from one nucleosome. In Ref. (21) a statistical mechanics model which constructed a nucleosome-DNA binding free energy based on the assumption of a strong DNA-histone interaction at points where the minor groove contacts the protein could reproduce the observed distribution of (positive and negative) linker lengths – as DNA unwraps there is an energy increase because hydrogen bonds are broken, but at the same time the entropy of the unwrapped section increases, leading to an oscillatory free energy. This model (which does not have a sequence dependent component) also explains the linker length periodicity through a DNA-nucleosome free energy profile which extends beyond the 147 bp footprint – this could arise through DNA-histone tail interactions, steric interactions between neighbouring nucleosomes (the bp separation of nucleosomes determines their relative orientation), or interactions with other proteins such as H1 linker histone (HHO1p in yeast). Intriguingly if we use the NucPosSimulator software with the chemical cleavage pile-up data as an input to obtain the most likely nucleosome positions, the linker periodicity is lost; this is despite the other reported trends being retained, and the positions of over 80% nucleosome centres being with 40 bp of those of the "unique map" obtained from peak finding. NucPosSimulator assumes a "hard" steric nucleosome-nucleosome interaction, and gives similar linker distributions even if the nucleosome footprint is reduced to allow overlaps; this suggests that a "soft" nucleosome-nucleosome interaction would be required to retain the linker periodicity (as implied by Ref. (21)). At any rate, a highly irregular pattern of linker lengths is prevalent across all of these nucleosome mapping methods. Furthermore, we do not expect the periodicity in linker lengths to affect our results, since the small (<5bp) changes in nucleosomes positions are below the resolution of our coarse-grained model (where DNA is represented by a chain of beads with size $\sim$ 7.35 bp).

11. Domain patterns can also be predicted analytically

The observation that nucleosome positions so strongly predict domain structure implies that the properties of chromatin fibres in yeast are highly dependant on the linker DNA between nucleosomes. To show this more explicitly we use some analytical concepts from polymer physics. First, we imagine taking our model chromatin fibre, and replacing all nucleosome beads with DNA beads, such that the chain is completely homogeneous. We then consider generating a contact map, but only using the beads which were originally nucleosomes; rather than doing a simulation, we instead assume that the level of interaction between beads i and $i+s$ has a power-law dependence $\sim s^{-1}$ (using an exponent close to that measured in Fig. S8). This reveals a domain pattern strikingly similar to the MicroC data (Fig. S13; actually using a different exponent, e.g. for a random or self-avoiding walk, gives similar results). The presence of DNA wrapped into nucleosomes means that the chromatin fibre has real domains of increased self-interaction. Additionally, in experimental and simulation (but not analytical) maps, domains persist when interactions are scaled by the "expected interactions" as common in HiC (Fig. S13b). This shows that the heterogeneous physical structure of the fibre in the nucleosomes and nucleosome depleted regions (present in experiments and simulations, but not represented in the analytics) leads to additional enrichment of contacts within a domain.

We can use this analytical approach to examine different patterns of linker lengths, to understand how these might lead to domains. For example, a possible chromatin structure might be for nucleosomes to be spaced regularly, but with occasional long linkers (nucleosome depleted regions or NDRs). Fig. S13c shows a map generated assuming nucleosomes are spaced with $\sim$ 30 bp linkers, with occasional $\sim$ 100 bp gaps (these lengths might be inferred from values of the nucleosome repeat length commonly quoted in the literature); we observe only a very weak domain-like pattern. The pattern can be strengthened by placing the regularly spaced nucleosomes closer together and increasing the length of the NDRs. However these domains look uniform and featureless compared to the pattern obtained with real nucleosome positions. From this we conclude that the observed domain patterns arise due to the whole distribution of long and short linkers – the former leading to boundaries, and the latter further increasing interactions with the domains.

12. Cell-to-cell variability in nucleosome positions

Above and in the main text we have detailed simulations where a single set of nucleosome positions is used for each region investigated. This was the set of "most likely" nucleosome positions generated from MNase data using the NucPosSimulator software. Here we investigate the effect of cell-to-cell variability of nucleosome positions. NucPosSimulator can also generate an ensemble of nucleosome position profiles which are consistent with the MNase data (see Ref. (2) for details; the software generates a single "most likely" set of positions

when used in "simulated annealing" mode, or an ensemble of configurations by default). By varying the parameters used by the software the level of variation between sets of positions can be controlled, and one might expect this to affect the output. Here we consider two cases: high and low variability. In both cases the mean linker length is kept consistent with values quoted in the literature (e.g. see Fig. S2c).

In Fig. S14a 20 sets of nucleosome positions (green) are shown alongside the "most likely" positions (blue) and the MNase-seq data from which these are obtained: this is the high variability case. We run a simulation for each set, and contact maps (where each row or column represents one nucleosome) generated from three of these are shown in Fig. S14b. Since there can be a different number of nucleosomes in each set of positions, the contact maps have different sizes, and so are not aligned vertically. Nevertheless it can be seen that some domains or boundaries are present across all three examples, whereas others are not. Since each map comes from a single simulation (averaged over time) they are noisier than a map generated from several simulations. To see how simulation-to-simulation variation affects a combined contact map, we must first convert the nucleosome-nucleosome based interactions into bp based interactions (since there is no one-to-one mapping of nucleosomes between the simulations). The top panel in Fig. S14c shows this combined map, with the other panels showing similar bp based maps from the MicroC data and a set of simulations based on the "most likely" nucleosome positions. We note here the poor agreement between the "cell-to-cell variation" simulations and the data (compared to the "most likely" simulations): the interaction map does not show clear domains.

In Fig. S15 similar results are shown, but here there is much lower variability between the different sets of nucleosome positions. In Fig. S15c there is clearly a much better visual agreement between the simulations (top) and data (middle); in fact the visual agreement is arguably better than that of the "most likely nucleosome position" simulations (bottom). This result suggests that in reality for yeast there is a low level of variability between cells within a population.

A number of new experimental methods for probing nucleosome position in singe cells are currently being developed (22, 23), and as more data becomes available in the future, it would be interesting to further investigate domain formation in single cells and how cell-to-cell variation affects population measures of 3-D structure. It would also be interesting to study cell-to-cell variation in other organisms, since, for example, nucleosome spacing and cell-to-cell variability of positions is expected to be much higher in higher eukaryotes.

References

1. Dang W, et al. (2014) Inactivation of yeast Isw2 chromatin remodeling enzyme mimics longevity effect of calorie restriction via induction of genotoxic stress response. *Cell Metabolism* 19:952–966.
2. Schöpflin R, et al. (2013) Modeling nucleosome position distributions from experimental nucleosome positioning maps. *Bioinformatics* 29:2380–2386.
3. Chen K, et al. (2013) Danpos: Dynamic analysis of nucleosome position and occupancy by sequencing. *Genome Research* 23:341–351.
4. Lee W, et al. (2007) A high-resolution atlas of nucleosome occupancy in yeast. *Nature Genetics* 39:1235.
5. Shivaswamy S, et al. (2008) Dynamic remodeling of individual nucleosomes across a eukaryotic genome in response to transcriptional perturbation. *PLOS Biology* 6:1–13.
6. Lantermann AB, et al. (2010) Schizosaccharomyces pombe genome-wide nucleosome mapping reveals positioning mechanisms distinct from those of saccharomyces cerevisiae. *Nature Structural & Molecular Biology* 17:251.
7. Hsieh TH, Fudenberg G, Goloborodko A, Rando O (2016) Micro-C XL: assaying chromosome conformation from the nucleosome to the entire genome. *Nature Methods* 13:1009.
8. Hsieh TH, et al. (2015) Mapping nucleosome resolution chromosome folding in yeast by micro-c. *Cell* 162:108–119.
9. Weiner A, et al. (2015) High-resolution chromatin dynamics during a yeast stress response. *Molecular Cell* 58:371–386.
10. Lopez-Serra L, Kelly G, Patel H, Stewart A, Uhlmann F (2014) The scc2-scc4 complex acts in sister chromatid cohesion and transcriptional regulation by maintaining nucleosome-free regions. *Nature Genetics* 46:1147.
11. Brogaard K, Xi L, Wang JP, Widom J (2012) A map of nucleosome positions in yeast at base-pair resolution. *Nature* 486:496.
12. Brackley CA, Cates ME, Marenduzzo D (2012) Facilitated diffusion on mobile dna: Configurational traps and sequence heterogeneity. *Physics Review Letters* 109:168103.
13. Brackley CA, Taylor S, Papantonisc A, Cook PR, Marenduzzo D (2013) Nonspecific bridging-induced attraction drives clustering of dna-binding proteins and genome organization. *Proceedings of the National Academy of Sciences USA* 110:E3605–E3611.
14. Brackley CA, Morozov AN, Marenduzzo D (2014) Models for twistable elastic polymers in brownian dynamics, and their implementation for lammps. *Journal of Chemical Physics* 140:135103.
15. Luger K, Mäder AW, Richmond RK, Sargent DF, Richmond TJ (1997) Crystal structure of the nucleosome core particle at 2.8 Å resolution. *Nature* 389:251.
16. Plimpton S (1995) Fast parallel algorithms for short-range molecular dynamics. *Journal of Computational Physics* 117:1–19.
17. Hajjoul H, et al. (2013) High-throughput chromatin motion tracking in living yeast reveals the flexibility of the fiber throughout the genome. *Genome Research* 23:1829–1838.
18. Langmead B, Salzberg SL (2012) Fast gapped-read alignment with bowtie 2. *Nature Methods* 9:357.
19. Hughes AL, Rando OJ (2014) Mechanisms underlying nucleosome positioning in vivo. *Annual Review of Biophysics* 43:41–63.
20. Engeholm M, et al. (2009) Nucleosomes can invade dna territories occupied by their neighbors. *Nature Structural & Molecular Biology* 16:151.
21. Chereji RV, Morozov AV (2014) Ubiquitous nucleosome crowding in the yeast genome. *Proceedings of the National Academy of Sciences USA* 111:5236–5241.
22. Small EC, Xi L, Wang JP, Widom J, Licht JD (2014) Single-cell nucleosome mapping reveals the molecular

basis of gene expression heterogeneity. *Proceedings of the National Academy of Sciences USA* 111:E2462–E2471.
23. Lai B, et al. (2018) Principles of nucleosome organization revealed by single-cell micrococcal nuclease sequencing. *Nature* 562:281–285.
